# The C-terminal cysteines in zinc transporter 8 (ZnT8) form an independent epitope for autoantibody binding in newly diagnosed type 1 diabetes

**DOI:** 10.1093/cei/uxag050

**Published:** 2026-08-31

**Authors:** Claire L Williams, S Rokni, V Lampasona, C Brigatti, B T Gillard, K M Gillespie, A J K Williams, A E Long

**Affiliations:** Bristol Medical School, University of Bristol, Level 2, Learning and Research Building, Southmead Hospital, Bristol, UK; Bristol Medical School, University of Bristol, Level 2, Learning and Research Building, Southmead Hospital, Bristol, UK; San Raffaele Diabetes Research Institute, IRCCS San Raffaele Scientific Institute, Milan, Italy; San Raffaele Diabetes Research Institute, IRCCS San Raffaele Scientific Institute, Milan, Italy; Bristol Medical School, University of Bristol, Level 2, Learning and Research Building, Southmead Hospital, Bristol, UK; Bristol Medical School, University of Bristol, Level 2, Learning and Research Building, Southmead Hospital, Bristol, UK; Bristol Medical School, University of Bristol, Level 2, Learning and Research Building, Southmead Hospital, Bristol, UK; Bristol Medical School, University of Bristol, Level 2, Learning and Research Building, Southmead Hospital, Bristol, UK

**Keywords:** autoantibodies, zinc transporter 8, site-directed mutagenesis, radiobinding assay, type 1 diabetes

## Abstract

Autoantibodies to zinc transporter 8 (ZnT8A) are important diagnostic and predictive markers for type 1 diabetes (T1D). Current methods to detect ZnT8A use C-terminal ZnT8 (aa268-369). Previous research identified three major ZnT8A epitopes (R325, W325, and a four-residue conformational epitope [R332/E333/K336/K340]), but the human-specific cysteine region has not been investigated. We sought to examine ZnT8A binding to three C-terminal cysteines and confirm previously known ZnT8A epitopes. Assay buffer constituent (Tween-20) and site-directed mutagenesis of radiolabelled ZnT8 antigen were utilized to compare ZnT8A binding in R325-ZnT8 and W325-ZnT8 monomeric radiobinding assays (RBAs). Truncated ZnT8 (D360X) and cysteine-to-serine mutations (C361S, C364S, and C368S) were compared with wild-type R325-ZnT8/W325-ZnT8 RBAs in 71 newly diagnosed T1D cases. Samples were indexed to controls and the median reduction in binding (MRB, %) was compared using Wilcoxon signed-ranked tests. Unbiased hierarchical clustering analysis was used to review effects of all ZnT8 mutations. A higher Tween-20 concentration reduced ZnT8A binding (*P* < 0.0001). All cysteine region mutations reduced 325-agnostic ZnT8A binding towards wild-type R325-ZnT8 (MRB range −37.0 to −66.4%, *P* < 0.0001) and W325-ZnT8 (MRB range −32.1 to −43.8%, *P* < 0.0001). Mutating the cysteine region nearly abolished binding (>75%) in 7.7–36.8% of R325-ZnT8/W325-ZnT8 positive ZnT8A, and in 10.5–13.2% of subjects studied, reduced binding below that of major ZnT8A epitopes. The cysteine region of C-terminal ZnT8 constitutes an independent epitope for ZnT8A, but mature ZnT8A responses in newly diagnosed T1D are heterogeneous. Epitope spreading of ZnT8A during the natural history of preclinical T1D remains unclear and requires further investigation in larger studies.

## Teaser

Autoantibodies to zinc transporter 8 (ZnT8A) are important diagnostic and predictive markers for type 1 diabetes (T1D). Current methods to detect ZnT8A use C-terminal ZnT8 (aa268-369) and previous research identified three major ZnT8A epitopes [R325, W325, and a four residue confirmational epitope (R332/E333/K336/K340 (REKK))], but the human-specific cysteine region has not been investigated. In this article, we confirm previous findings of R325/W325/REKK epitopes, show that a high Tween-20 concentration reduces ZnT8A binding, and demonstrate that mutating individual cysteines in C-terminal ZnT8A reduces binding in 325-agnostic ZnT8A and in at least 10% of T1D cases reduced binding below that of previously described epitopes.

## Graphical Abstract

**Graphical Abstract:**
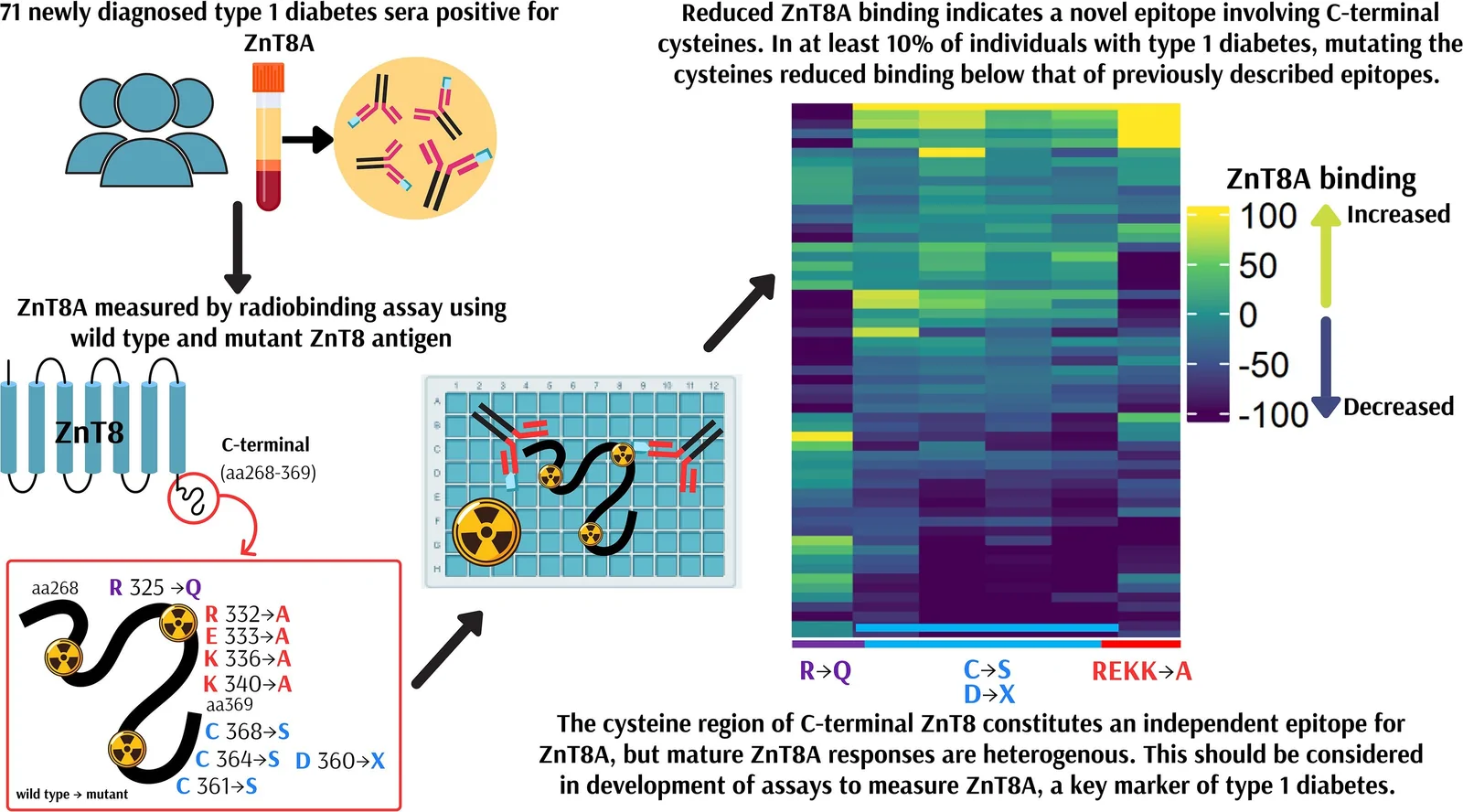

## Introduction

Identified in 2007, zinc transporter 8 (ZnT8) is a major autoantigen in type 1 diabetes (T1D) that is almost exclusively localized to insulin secretory granules of pancreatic beta cells [1, 2]. Many studies have since found that autoantibodies to ZnT8 (ZnT8A) are present in ∼60–80% of individuals with new-onset T1D [1, 3]. In combination with islet autoantibodies to insulin (IAA), glutamic acid decarboxylase 65 (GADA), and islet antigen 2 (IA-2A), up to >90% of new-onset T1D can be identified and are the principal biomarkers for predicting future risk of the disease [3–5].

Encoded by *SLC30A8*, ZnT8 is a 369-amino acid polytopic transmembrane protein comprised of six transmembrane domains, a luminal-facing histidine-rich loop, eight exon regions, and cytoplasmic –NH_2_ (N-) and –COOH (C-) terminal ends [6, 7]. *In vivo*, ZnT8 forms a dimeric structure from two full-length ZnT8 monomers [8]. Due to the probability that ZnT8A cannot access the embedded transmembrane regions that make up half of ZnT8, monomeric or fusion ZnT8 antigen segments of the cytosolic and luminal regions have been utilized to study the ZnT8 humoral response in T1D [1, 9, 10]. In newly diagnosed T1D, the major regions of ZnT8 that mature ZnT8A recognize are C-terminal, accounting for 80–90% of ZnT8A reactivity while only ∼10% recognize the N-terminal [1, 9].

Within the C-terminal of human ZnT8, a common single nucleotide polymorphism (SNP) in *SLC30A8*, rs13266634 (C/T; minor allele frequency 0.3), causes a non-synonymous arginine (R) to tryptophan (W) modification at amino acid 325 (R325W) [11, 12]. A second SNP rs16889462 (G/A) at the same position encodes glutamine (Q) has also been identified, but is rare in European populations (<1%) and is present in ∼9% of African-American populations [10, 12]. A genome-wide association study (GWAS) found that the CC genotype (R325) confers minor risk of type 2 diabetes but in T1D, the rs13266634 SNP forms two major epitopes of ZnT8A. This polymorphism influences the type of ZnT8A a person produces, which can be categorized into three ZnT8A specificities; R325-specific (ZnT8RA), W325-specific (ZnT8WA), and 325-agnostic ZnT8A that bind ZnT8 independent of this polymorphism [9, 13, 14]. This demonstrates that individual responses to endogenous ZnT8 protein are determined by genotype. However, ∼70% of ZnT8A in T1D cases produce 325-agnostic ZnT8A, which can be determined using the naturally occurring Q325 variant (Q325-ZnT8; ZnT8QA) without compromising the structural integrity of ZnT8 [9].

Following the observation that human ZnT8A from new-onset T1D subjects do not recognize murine C-terminal ZnT8, despite ∼80% homology (80/102 residues), a study found that independent of aa325, residues R332, E333, K336, and K340 (REKK) formed a conformational epitope [10]. The presence of conformational epitopes within the C-terminal of ZnT8 has been further supported by the observations that linear 15-aa C-terminal ZnT8 peptides were insufficient to displace ZnT8A [13], and short (aa318-331) and long (aa268-369) ZnT8 peptides could not displace R325-specific and W325-specific ZnT8A [14]. This suggests other non-polymorphic conformational epitopes are important for ZnT8A responses.

Cysteines enable tertiary structure formation of proteins and are thought integral to conformational epitopes of humoral responses. Modification of the core cysteines C909 and C945 in IA-2/IA-2β antigens *in vitro*, respectively, reduced IA-2A binding in newly diagnosed T1D sera detected by radiobinding assay (RBA) [15]. Additionally, concentrations of common buffer constituents used in immunoassays, such as the non-ionic surfactant Tween-20, were shown to influence IA-2A binding in an epitope-related manner, and this has important connotations for autoantibody measurement and inter-laboratory concordance [16, 17]. To date, antigen-specific RBAs are well described and remain widely used for islet autoantibody measurement. For immunoprecipitating ZnT8A in serum by RBA, individual laboratories utilize monomeric (R325-ZnT8 or W325-ZnT8) or heterodimeric (R325-ZnT8 + W325-ZnT8) [^35^S]-methionine labelled ZnT8 peptides [18]. To our knowledge, the importance of the three C-terminal cysteines (C361, C364, and C368) in ZnT8 and Tween-20 buffer concentration used in RBAs have not been explored for ZnT8A measurement. Intriguingly, two of the three C-terminal cysteines [C361 and C364 in the highly conserved CXXC motif that’s specific for vesicular zinc transporters (2/3/4/8)] have recently been shown to have high affinity for zinc and may aid in the allosteric mechanism of ZnT8 required for zinc trafficking into insulin secretory granules of pancreatic beta cells [19–21]. This human-specific cysteine-rich region in ZnT8 may have important implications for both assay performance, stabilizing protein structure, and ZnT8A epitope recognition.

In this study, we sought to investigate the importance of the C-terminal cysteines in ZnT8 for ZnT8A binding in newly diagnosed T1D sera by RBA through determining the effect of Tween-20 concentration, single/double cysteine-to-serine mutations, and a truncation at aa360 (D360X). We also aimed to confirm and evaluate the cysteine region according to the major epitopes of ZnT8A (R325, W325, and REKK) through Q325, *SLC30A8* genotype, and REKK-to-alanine mutations.

## Materials and methods

### Newly diagnosed T1D study subjects

#### Subjects from the Southwest of England New-Diagnosed Collection

The Southwest of England New-Diagnosed Collection (SWENDIC) study comprises individuals with newly diagnosed T1D recruited from 14 centres in South-West England. Anonymized serum samples from 106 cases taken <1 year from diagnosis were selected for Tween-20 concentration experiments [median age 25 years (range 17–41); median duration 26 days (range 5–322)]. Use of these anonymized samples is currently approved by Wales Research Ethics Committee 6 (Swansea) (reference: 19/WA/0295).

#### Subjects from the Bart’s Oxford family study

The well-characterized population-based Bart’s Oxford (BOX) family study, established in 1985, has recruited and prospectively followed individuals under 21 years of age with newly diagnosed T1D and their FDRs within the former Oxford Regional Health Authority in the UK [22]. The BOX study is currently approved by South Central—Oxford C. Research Ethics Committee (reference: 15/SC/0310). Participants provided informed, written consent and the study was performed according to the principles of the Declaration of Helsinki. This cohort was used to investigate ZnT8A binding to the cysteine-rich region of ZnT8 and other previously identified polymorphic and non-polymorphic epitope regions.

Serum samples taken within 3 months of T1D diagnosis from BOX participants were selected based on available historical data from monomeric (R325-ZnT8/W325-ZnT8) RBAs and sufficient sample volume for C-terminal ZnT8/ZnT8A characterization [*n* = 71; 43 male (60.6%); median age at onset 9.1 years (range 1.9–19.3); 61 (85.9%) were sampled within 2 weeks of diagnosis; 25 (35.7%) had the high T1D risk HLA class II genotype (*DR3-DQ2/DR4-DQ8*)] (Table 1).

**Table 1:** Cohort description of selected newly diagnosed T1D subjects from the BOX study.

Variable	Number (%)
Gender (*n* = 71)	
Male	43 (60.6)
Female	28 (39.4)
Age at onset (*n* = 71)	
0–5 years	12 (16.9)
5–10 years	25 (35.2)
10–15 years	22 (31.0)
15–20 years	12 (16.9)
Autoantibody (*n* = 71)	
IAA (*n* = 61)*	49 (80.3)
GADA (*n* = 71)	57 (80.3)
IA-2A (*n* = 71)	61 (85.9)
ZnT8A (*n* = 71)	71 (100.0)
ZnT8RA	57 (80.3)
ZnT8WA	53 (74.6)
HLA Class II (*n* = 70)^a^	
High	25 (35.7)
Moderate	36 (51.4)
Low	9 (12.9)
HLA Class I	
HLA-A*24 negative (*n* = 66)	54 (81.8)
HLA-B*18 negative (*n* = 65)	55 (84.6)
HLA-B*39 negative (*n* = 65)	59 (90.8)
*SLC30A8* genotype (*n* = 69)**	
CC	30 (43.5)
CT	27 (39.1)
TT	12 (17.4)

^a^High (DR3-DQ2 and DR4-DQ8), medium (presence of either DR3-DQ2 or DR4-DQ8 with a non-protective HLA haplotype), neutral (presence of either DR3-DQ2 or DR4-DQ8 with a protective haplotype or haplotypes not associated with type 1 diabetes) and low (presence of one or two protective haplotypes in the absence of DR3-DQ2 or DR4-DQ8) HLA susceptibility/protective haplotypes, which was determined as described previously [23]. All subjects were selected from the BOX study.

^*^61/71 (85.9%) of the cohort was sampled within 2 weeks of diagnosis to accurately determine IAA positivity to endogenous insulin prior to insulin treatment [24].

^**^ *SLC30A8* genotype (rs13266634); underlined TT genotype denotes the minor allele.

Abbreviations: IA-2A, islet antigen 2 autoantibodies; IAA, insulin autoantibodies; GADA, glutamic acid decarboxylase 65 autoantibodies. Zinc transporter 8 autoantibodies (ZnT8A) positive for R325 (ZnT8RA) and W325 (ZnT8WA) ZnT8 variants, HLA (human leucocyte antigen).

### Site-directed mutagenesis of ZnT8 antigen constructs

The pCMVTnT vector (Promega, WI, USA) containing wild-type C-terminal (aa268-369) monomeric R325-ZnT8 or W325-ZnT8 were kind gifts of V. Lampasona (Milan, Italy; Supplementary Figure S1). SDM to appropriately replace selected amino acids with serine or alanine was performed according to Agilent (Santa Clara, CA, USA) QuickChange II SDM Instruction Manual. PAGE-purified designed primers of single mutations were used (Sigma-Aldrich, Dorset, UK; Supplementary Table S1).

Plasmids were transformed into chemically competent DH5α *Escherichia coli* cells (Invitrogen, MA, USA). To confirm the success of the mutations, plasmid DNA was prepared according to the QIAprep Spin Miniprep Protocol (Qiagen, Hilden, Germany) and sequenced by MWG Eurofins Genomics (Ebersberg, Germany) using a standard SP6 primer. Plasmids containing single mutations were subsequently used as template DNA for further SDM using primers designed to generate plasmids with multiple mutations (Supplementary Table S1). The truncation at aa360 (D360X) was generated by V. Lampasona’s laboratory. A ZnT8 mutant antigen, encoding murine REKK [T332, G333, Q336, deletion(-)340; TGQ-], was generated through molecular cloning strategies and was assessed in a subset of the cohort (*n* = 12/71) to compare to the REKK-A ZnT8 mutant antigen to inform structural antigen integrity. A total of 13 mutant ZnT8 constructs were generated and evaluated in the BOX study cohort according to ZnT8A specificity towards wild-type R325-ZnT8/W325-ZnT8.

### Detection of ZnT8A by RBA

Recombinant monomeric wild-type (R325-ZnT8/W325-ZnT8; aa268-369) and mutant ZnT8 antigens were expressed and labelled with L-[^35^S]-methionine (Revvity, Waltham, MA, USA) by an *in vitro* transcription/translation reticulocyte lysate system using SP6 polymerase (Promega, Madison, WI, USA). Autoantibodies to wild-type and mutant ZnT8 were detected by RBA, as described previously [25]. To account for intra- and inter-assay variation, radiolabelled ZnT8 antigens and the panel of ZnT8A positive serum samples were prepared and tested in parallel against mutated and wild-type ZnT8 antigens to compare ZnT8RA/ZnT8WA binding under the same experimental conditions and within the same assay run. Additionally, positive (R325-specific, W325-specific, and 325-agnostic) and negative controls were included on each assay plate to account for inter-assay variation and were utilized to index samples for analysis. Raw data were expressed in counts per minute (CPM) and mean CPM between duplicates was used for analysis.

An in-house logarithmic standard curve made from 8 serial dilutions of a high T1D pool of ZnT8RA/ZnT8WA positive sera was used to determine wild-type ZnT8A binding expressed as arbitrary units (AU). The positivity threshold was set at the 97.5th percentile of 523 healthy schoolchildren, described previously [26]. In the 2024 Islet Autoantibody Standardization Programme (IASP2024) workshop, the adjusted sensitivity at 95% specificity (AS95) of R325-ZnT8 RBA and W325-ZnT8 RBA was 78.0% and 70.0%, respectively. Informed by previous investigations of another T1D humoral response towards a transmembrane protein (IA-2A/IA-2) [15], high (1%) and low (0.15%; standard) Tween-20 assay buffer concentrations on ZnT8A binding (mean CPM) were compared in both wild-type monomeric RBAs.

### *SLC30A8* genotype determination

To investigate the influence of ZnT8A specificity towards wild-type and mutant ZnT8 antigens, the *SLC30A8* SNP (rs13266634) was determined where genetic samples were available using a Taqman SNP kit following the manufacturer’s instructions (ThermoFisher). The BOX study had available genetic samples for *SLC30A8* genotyping for subsequent analysis [*n* = 69/71 (97.2%); Table 1].

### Data transformation and statistical analysis

**Figure 1:**
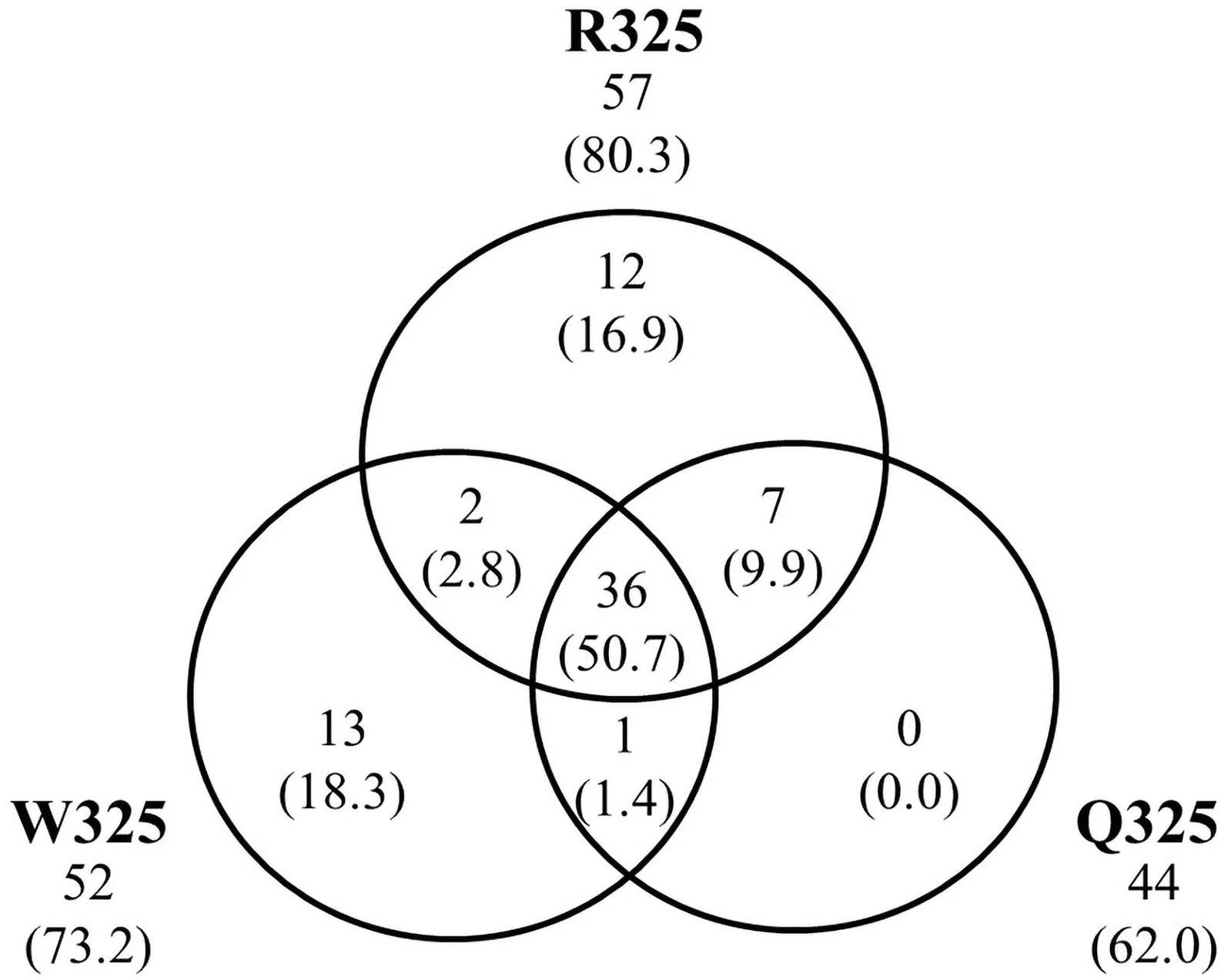
Categorization of ZnT8A according to the major polymorphic epitope (325) in newly diagnosed T1D subjects. ZnT8A positivity/specificity profiles according to the major polymorphic epitope region [R325 (80.3%), W325 (73.2%), and Q325 (62.0%) wild-type ZnT8 antigen] in 71 new-onset T1D subjects from the BOX study sampled within 3 months of diagnosis [43 males (60.6%); median age at onset 9.1 years (range 1.9–19.3)]. Of 71 subjects, 12 (16.9%) samples had R325-specific, 13 (18.3%) had W325-specific, and 46 (64.8%) had 325-agnostic ZnT8A responses, exhibiting positivity towards ≥2 wild-type ZnT8 variants with 36 (50.7%) positive for all three variants. Due to the pre-selection of the cohort based on R325-ZnT8/W325-ZnT8 positivity by respective monomeric RBAs, there was no detection of Q325-specific responses, but these are rare based on previous studies [10, 12]. For the remainder of analysis, sera that were positive towards R325-ZnT8/W325-ZnT8 (*n* = 2), R325-ZnT8/Q325-ZnT8 (*n* = 7), and W325-ZnT8/Q325-ZnT8 (*n* = 1) wild-type antigens were classified were classified as 325-agnostic ZnT8A responses.

Spearman’s rank (*r*) correlation test was used to compare ZnT8A binding to wild-type ZnT8 antigens (AU) and mutant ZnT8 antigens (mean cpm). Proportions of categorical variables were compared using Chi-squared (χ^2^) or Fisher’s exact tests where appropriate. Data were categorized by ZnT8A specificity to wild-type ZnT8 (R325-specific/W325-specific/325-agnostic) once determined (Fig. 1). Of 71 newly diagnosed T1D BOX subjects, 57 (80.3%), 52 (73.2%), and 44 (62.0%) had ZnT8A positivity towards wild-type ZnT8 encoding R325, W325, and Q325, respectively. Overall, 12 (16.9%) subjects had R325-specific, 13 (18.3%) had W325-specific, and 46 (64.8%) had 325-agnostic ZnT8A responses, exhibiting positivity towards ≥2 wild-type ZnT8 variants. A total of 36 (50.7%) subjects were positive for all three wild-type variants. For cysteine mutations, the reduction in ZnT8A binding (RB, %) of each ZnT8 mutant for analysis was determined by indexing T1D samples to positive controls, corresponding to the ZnT8A specificity of each sample (R325-specific, W325-specific, 325-agnostic), in both wild-type and mutant ZnT8 antigens separately in the same assay run [(mean cpm of sample (S) − negative control) ÷ (S − positive control) × 100 (%)] and calculating the difference ((mutant index − wild-type index) ÷ wild-type index × 100 (%)). To ascertain the effect of the polymorphic 325 epitope, a 325-agnostic control was used for indexing samples whereas for REKK, R325-specific and W325-specific controls were utilized for indexing samples. One-sample Wilcoxon tests were used to evaluate whether the median RB (MRB, %) towards mutant ZnT8 differed from wild-type ZnT8. For Q325 and REKK-A mutations, one-way Kruskal–Wallis (*H*) with Dunn post-hoc tests for multiple comparisons were used to evaluate the effect of mutations on ZnT8A binding according to categories of ZnT8A specificity as the same index control(s) were used. To evaluate and compare ZnT8A responses to all investigated mutant ZnT8 antigens in all samples in the cohort, visual heatmaps for R235-reactive and W325-reactive ZnT8A responses were generated using the ComplexHeatmap [27] package with Circlize [28] and viridis [29] in R [30]. The Heatmap function uses complete unbiased hierarchical clustering with Euclidean distances to group sera based on similarity of ZnT8A binding to each ZnT8 antigen investigated. No scaling was used as all RB values were in percentages. Unless otherwise stated, all statistical analysis was performed in GraphPad PRISM (GraphPad Software, Inc., CA, USA; v. 9.1.0), and *P* < 0.05 was considered significant in all analyses.

## Results

### The effect of Tween-20 on ZnT8A binding

Of 106 newly diagnosed T1D subjects from SWENDIC, 63 (59.4%) were positive for ZnT8A; 57 (53.8%) R325-positive and 47 (44.3%) W325-positive. Of 63, 16 (25.4%) were R325-specific, 6 (9.5%) were W325-specific, and 41 (65.1%) were 325-agnostic. The effect of increasing the Tween-20 concentration from 0.15% (standard) to 1% in TBST assay buffer resulted in an MRB of −27.4% in R325-positive and −61.6% in W325-positive (both *P* < 0.0001, Supplementary Figure S2), and there was a marked variability among the sera (MRB range −136.0% to +91.6%). In 325-agnostic ZnT8A responses, the MRB of W325-positive sera was greater by −18.5% than R325-positive sera (*P* < 0.0001). Overall, ZnT8A binding was nearly abolished (>−75% MRB) in 13/57 (22.8%) and 17/47 (36.2%) of R325-positive and W325-positive sera, respectively. This suggests that 1% Tween-20 can alter the conformational structure of ZnT8 *in vitro* and disrupt major ZnT8A epitopes, but the degree of this effect differs between sera. For further experiments, TBST with 0.15% Tween-20 was used.

### The effect of mutating C361, C364, and C368 to serine on ZnT8A binding

#### Single cysteine-to-serine mutations

**Figure 2:**
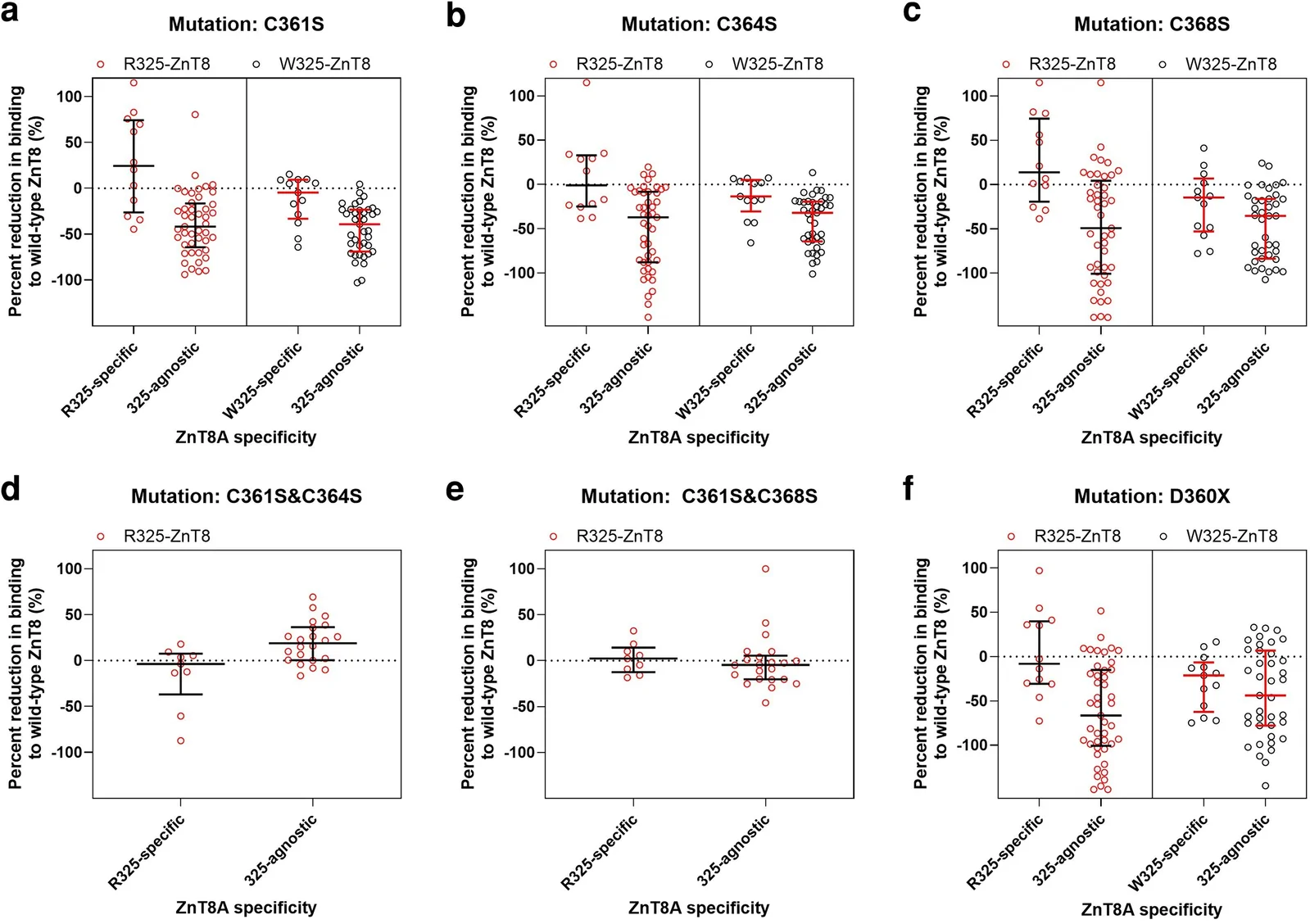
The effect of mutating or truncating the cysteine-rich region of C-terminal ZnT8 according to ZnT8A specificity. Black dotted line: no change in ZnT8A binding compared with wild-type ZnT8 antigen. Red/black bars denote median and interquartile ranges. The reduction in ZnT8A binding was capped at +115% if ≥+120% and capped at −150% if ≥−150% for resolution. In all ZnT8A responses, single cysteine-to-serine mutations (a–c), and truncating the cysteine-rich region of the C-terminal (f) did not significantly impact R325-specific or W325-specific responses (all *P* > 0.05), but in all 325-agnostic responses, single cysteine mutations and the D360X truncation significantly reduced ZnT8A binding from zero (all *P* < 0.0001). Double cysteine-to-serine mutations (d and e) did not impact R325-specific responses (*P* > 0.05), but in 325-agnostic ZnT8A responses, C361S/C364S caused an increase in ZnT8A binding (*P* = 0.0008), which was not observed for C361S&C368S (*P* > 0.05). In all mutations to the cysteine-rich region of C-terminal ZnT8, there were marked differences in ZnT8A binding between newly diagnosed T1D sera, regardless of the specificity of the ZnT8A response.

In 71 newly diagnosed T1D subjects from the BOX study, single cysteine-to-serine mutations C361S, C364S, and C368S caused significant reductions in binding in 325-agnostic ZnT8A in both R325-positive (*n* = 45) and W325-positive (*n* = 39) responses (all *P* < 0.0001), but not in R325-specific (*n* = 12) or W325-specific (*n* = 13) responses (all *P* > 0.05, Fig. 2a–c). Whilst R325-specific and W325-specific responses were not significantly impacted overall, there were a few sera that were negatively impacted by the single cysteine mutations, particularly in W325-specific (MRB range −4.7% to −14.6%) compared to R325-specific (MRB range −0.9% to +24.4%) responses. Generally, the MRB in all 325-agnostic ZnT8A responses was comparable across the three single serine-to-cysteine mutations to both R325-ZnT8 (MRB range −37.0 to −49.3%, *P* < 0.0001) and W325-ZnT8 (range −32.1% to −39.2%, *P* < 0.0001) antigens (Table 2). The raw ZnT8A binding (mean cpm) of all responses to mutant ZnT8 antigens at the cysteine sites were highly correlated with wild-type R325-ZnT8/W325-ZnT8 antigens (*r* range 0.85–0.98, *P* = 0.0003–<0.0001; Supplementary Figure S3), but there was evidence of heterogeneity across the cohort in all cysteine mutations across all ZnT8A responses and levels. In some samples, single cysteine-to-serine mutations improved ZnT8A binding [>0% MRB; *n* = 10–19 of 57 (17.5–33.3%) R325-positive and *n* = 7 of 53 (13.5%) W325-positive ZnT8A] and in other samples, ZnT8A binding was nearly abolished [>−75% MRB; *n* = 7–21 of 57 (12.3.5–36.8%) R325-positive; *n* = 4–15 of 53 (7.7–28.8%) W325-positive ZnT8A]. Collectively, this suggests the cysteine-region modifies ZnT8A binding differentially in different sera.

**Table 2:** The median reduction in binding and correlation between mutant ZnT8 (C361S, C364S, C368S, and D360X) and wild-type ZnT8 by ZnT8A specificity.

Wild-type ZnT8 antigen	ZnT8A specificity	*n*	ZnT8 mutation in cysteine region	Median reduction (%) in ZnT8A binding (range) δ	*P*-value*
R325	R325-specific	12	C361S	+24.4 (−44.6 to +115)	NS
			C364S	−0.9 (−38.4 to +115)	NS
			C368S	+13.8 (−38.7 to +115)	NS
			D360X	−8.1 (−72.8 to +97.2)	NS
		9Δ	C361S/C364S	−3.7 (−87.3 to +17.9)	NS
			C361S/C368S	+2.3 (−18.4 to +32.5)	NS
R325	325-agnostic	45§	C361S	−41.8 (−93.9 to +80.3)	<0.0001
			C364S	−37.0 (−150 to +19.8)	<0.0001
			C368S	−49.3 (−150 to +115)	<0.0001
			D360X	−66.4 (−150 to +51.7)	<0.0001
		22Δ	C361S/C364S	+18.9 (−16.5 to +69.1)	0.0008
			C361S/C368S	−4.6 (−45.8 to +100)	NS
W325	W325-specific	13	C361S	−4.7 (−64.2 to +15.3)	NS
			C364S	−13.5 (−66.0 to +7.3)	NS
			C368S	−14.6 (−78.0 to +41.3)	NS
			D360X	−21.2 (−74.8 to +16.7)	0.0046
W325	325-agnostic	39§	C361S	−39.2 (−102.9 o + 4.5)	<0.0001
			C364S	−32.1 (−101.2 to 13.3)	<0.0001
			C368S	−35.5 (−107.4 to +24.2)	<0.0001
			D360X	−43.8 (−145.8 to +33.1)	<0.0001

NS, not significant. Δ In a subset of the cohort (n = 31/71; 43.7%), double cysteine-to-serine mutations, C361S/C364S and C361S/C368S were investigated in R325-positive ZnT8A responses (*n* = 9 R325-specific; *n* = 22 325-agnostic). § denotes individuals that are positive for more than one wild-type ZnT8A variant (R325-ZnT8/W325-ZnT8/Q325-variant) and are classified as 325-agnostic. δ reduction in ZnT8A binding was capped at +115% if ≥+120% and capped at −150% if ≥−150%.

^*^Wilcoxon signed-rank test was used to evaluate change from zero in ZnT8A binding; all investigated single and the D360X mutations within the cysteine-rich region of C-terminal ZnT8 caused significant reductions in ZnT8A binding in 325-agnostic responses (*P*=<0.0001), but not R325-specific/W325-specific responses (*P* > 0.05). Double cysteine-to-serine mutations (C361S/C364S and C361S/C368S) did not cause significant changes to ZnT8A binding from zero, but there was an indication that C361S/C364S improved ZnT8A binding in 325-agnostic responses (*P* = 0.0008).

#### Double cysteine-to-serine mutations

In a subset of the cohort (*n* = 31/71; 43.7%), double cysteine-to-serine mutations, C361S/C364S and C361S/C368S were investigated in R325-positive ZnT8A responses (*n* = 9 R325-specific; *n* = 22 325-agnostic). Despite the effect of mutating individual cysteines, double cysteine-to-serine mutations did not cause a significant reduction in R325-positive ZnT8A binding (*P* > 0.05; Fig. 2d and e), but binding of 325-agnostic ZnT8A in C361S/C364S was greater than zero (*P* = 0.0008; Table 2). Therefore, there does not appear to be a sequential negative impact of mutating more than one C-terminal cysteine, and this was not investigated further.

### The effect of truncating at D360 (D360X) on ZnT8A binding

The D360X truncation removed all three C-terminal cysteines. This truncation reduced ZnT8A binding in W325-specific (*P* = 0.0046) and 325-agnostic responses to both R325-ZnT8 and W325-ZnT8 antigens (both *P* < 0.0001), but not R325-specific responses (*P* > 0.05, Fig. 2f). The raw ZnT8A binding (mean cpm) of all responses to the D360X ZnT8 antigen were highly correlated with wild-type R325-ZnT8/W325-ZnT8 antigens (*r* range 0.90–0.92, all *P* < 0.0001; Supplementary Figure S3), but there was evidence of heterogeneity across the cohort in all cysteine mutations across all ZnT8A responses and levels. In some samples, D360X improved ZnT8A binding [>0% MRB; *n* = 13 of 57 (22.8%) R325-positive and *n* = 13 of 53 (25.0%) W325-positive ZnT8A] and in other samples, ZnT8A binding was nearly abolished [>−75% MRB; *n* = 21 of 57 (36.8%) R325-positive; *n* = 12 of 53 (23.1%) W325-positive ZnT8A].

### Confirmation of previously identified ZnT8A epitopes

#### The polymorphic SNP site at amino acid 325

Encoding Q325 in wild-type ZnT8 (Q325-ZnT8) caused significant reductions in binding in R325-specific (*P* = 0.0005) and W325-specific (*P* = 0.0002) ZnT8A responses, but not 325-agnostic responses (*P* > 0.05). Similarly, the MRB demonstrates that Q325 abolishes (≥−90%) ZnT8A binding in R325-specific (MRB −99.3%) and W325-specific (MRB −98.5%) responses, compared to 325-agnostic responses to either R325-ZnT8 (MRB −5.1%) or W325-ZnT8 (MRB +1.7%) antigens, clearly demonstrating the range of effect according to ZnT8A specificity (*H* 55.0, *P* < 0.0001; Supplementary Figure S4), where all pairwise comparisons were significant between R325-specific/W325-specific and 325-agnostic ZnT8A responses (adj. *P* < 0.0001).

The specificity of ZnT8A responses towards wild-type ZnT8 variants was associated with *SLC30A8* genotypes (Supplementary Table S2). The presence of 325-agnostic ZnT8A, that respond to all R325/W325/Q325 wild-type variants, was highest in CT heterozygotes (*P* = 0.0006). Whereas R325-specific and W325-specific ZnT8A corresponded with genotypes containing the C allele (*P* = 0.013) and T allele (*P* = 0.0002), respectively.

#### The conformational epitope site REKK

**Figure 3:**
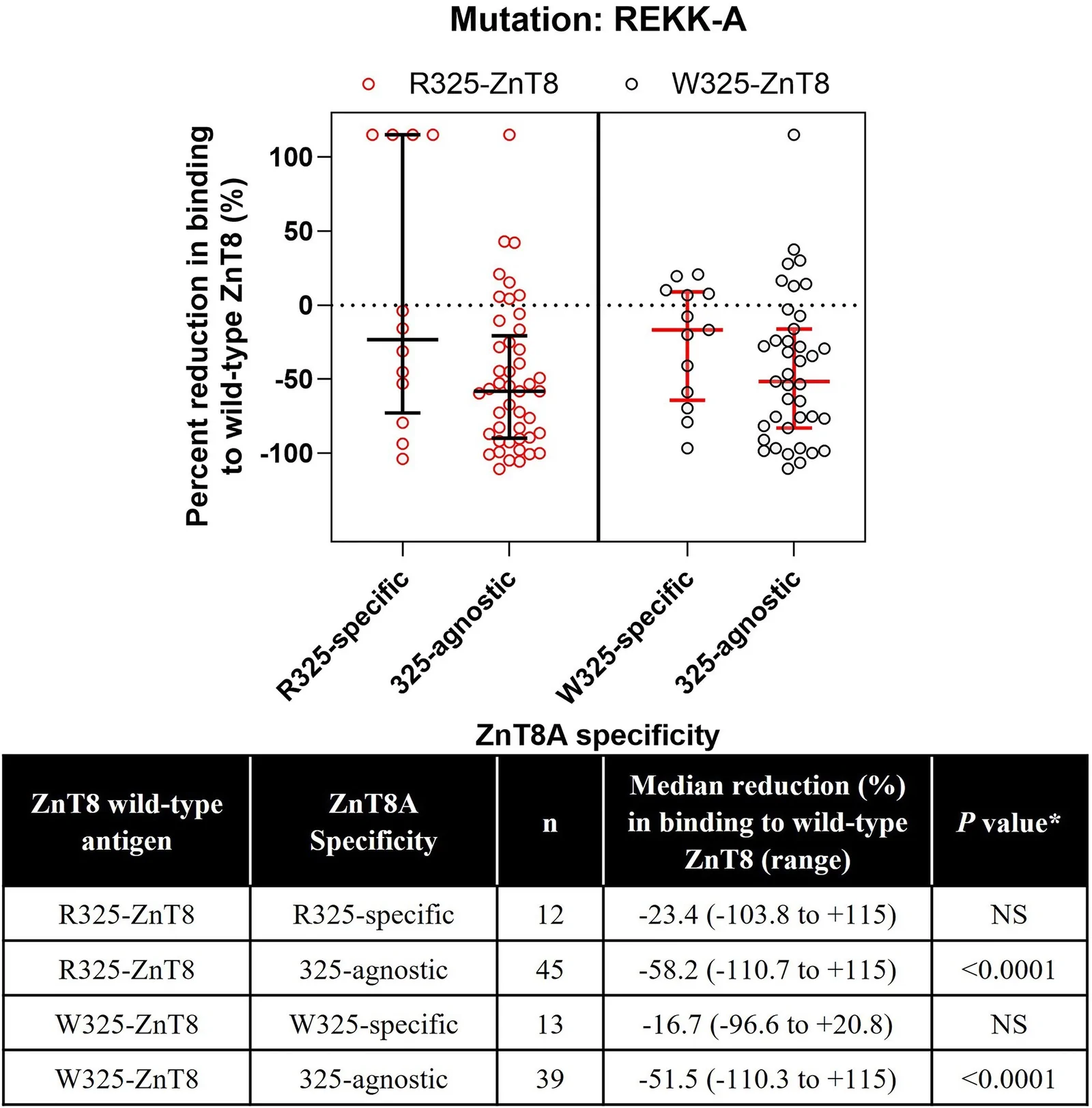
The effect of mutating REKK-to-alanine in C-terminal ZnT8 according to ZnT8A specificity. Black dotted line indicates no change in ZnT8A binding compared with wild-type R325-ZnT8/W325-ZnT8 antigens. Red/black bars denote median and interquartile ranges. The reduction in ZnT8A binding was capped at +115% if ≥+120% and capped at −150% if ≥ −150% for resolution. *Wilcoxon signed-rank test was used to evaluate change from zero in ZnT8A binding. The REKK-A mutation significantly reduced binding in 325-agnostic ZnT8A responses to R325-ZnT8/W325-ZnT8 (both *P* < 0.0001), but not R325-specific or W325-specific responses (both *P* > 0.05). There was marked differences in ZnT8A binding between newly diagnosed T1D sera, but there was no difference in the median reduction in ZnT8A binding according to ZnT8A specificity (Kruskal–Wallis *H* statistic 6.3; *P* = 0.0961; adj. *P* > 0.2930), when adjusted for multiple comparisons.

The REKK-A mutation caused significant reductions in binding in 325-agnostic ZnT8A responses to both R325-ZnT8 and W325-ZnT8 antigens (MRB range −51.5% to −58.2%, both *P* < 0.0001), but not R325-specific (MRB −23.5%) and W325-specific (MRB −16.7%) ZnT8A responses (both *P* > 0.05, Fig. 3). The MRB according to ZnT8A specificity did not differ when adjusted for multiple comparisons (*H* 6.3, *P* = 0.0961, adj.p > 0.2930). Nevertheless, there was a marked variability of the effect among the sera. In some samples, REKK-A improved ZnT8A binding [>0% MRB; *n* = 12 of 57 (21.1%) R325-positive and *n* = 12 of 53 (23.1%) W325-positive ZnT8A] and in other samples, ZnT8A binding was nearly abolished [>−75% MRB; *n* = 20 of 57 (35.1%) R325-positive; *n* = 17 of 53 (32.7%) W325-positive ZnT8A]. The heterogeneity of effects across the cohort caused by REKK-A is evident in the raw ZnT8A binding (mean cpm) across all ZnT8A responses and levels, despite the high correlation with wild-type ZnT8 antigens (Supplementary Figure S5).

Of 44 subjects with 325-agnostic ZnT8A that remained positive towards Q325-ZnT8 antigen and were positive for R325-ZnT8 and/or W325-ZnT8 wild-type antigens, the REKK-A mutant when Q325 was also present caused a MRB of −44.3% (*P* = 0.0054), regardless of positivity to two (*n* = 8, MRB −78.4%) or three (*n* = 36, MRB −29.4%) 325- ZnT8 antigen variants (*P* = 0.0801). This indicates that the REKK-A mutation causes comparable changes in 325-agnostic ZnT8A binding across all wild-type ZnT8 antigens (R325/W325/Q325), but the effect was not unanimous for all sera. In some samples, Q325-REKK-A improved ZnT8A binding [>0% MRB; *n* = 15 of 44 (34.1%)], and a comparable number of samples where Q325-REKK-A nearly abolished ZnT8A binding [>−75% MRB; *n* = 15 of 44 (34.1%)].

### Unbiased hierarchical cluster analysis showed that the cysteine-rich region of C-terminal ZnT8 forms a minor epitope for ZnT8A recognition that is independent of the polymorphic R325W and conformational REKK sites

The aim of this analysis was to identify patterns of reduced binding between the different epitopes. Specifically, we sought to understand whether (i) the cysteine mutants were independent i.e. impacted different sera compared with the known REKK or R325/W325/Q325 major epitopes, (ii) ZnT8A epitopes were exclusive i.e. a single sera could be impacted by multiple epitopes. To investigate this, unbiased hierarchical cluster analysis was performed, combining the existing experimental data from this study, to identify clusters of newly diagnosed T1D subjects based on the reduction in ZnT8A binding across all investigated C-terminal ZnT8 mutant antigens. This was performed separately for R325-positive and W325-positive responses.

**Figure 4:**
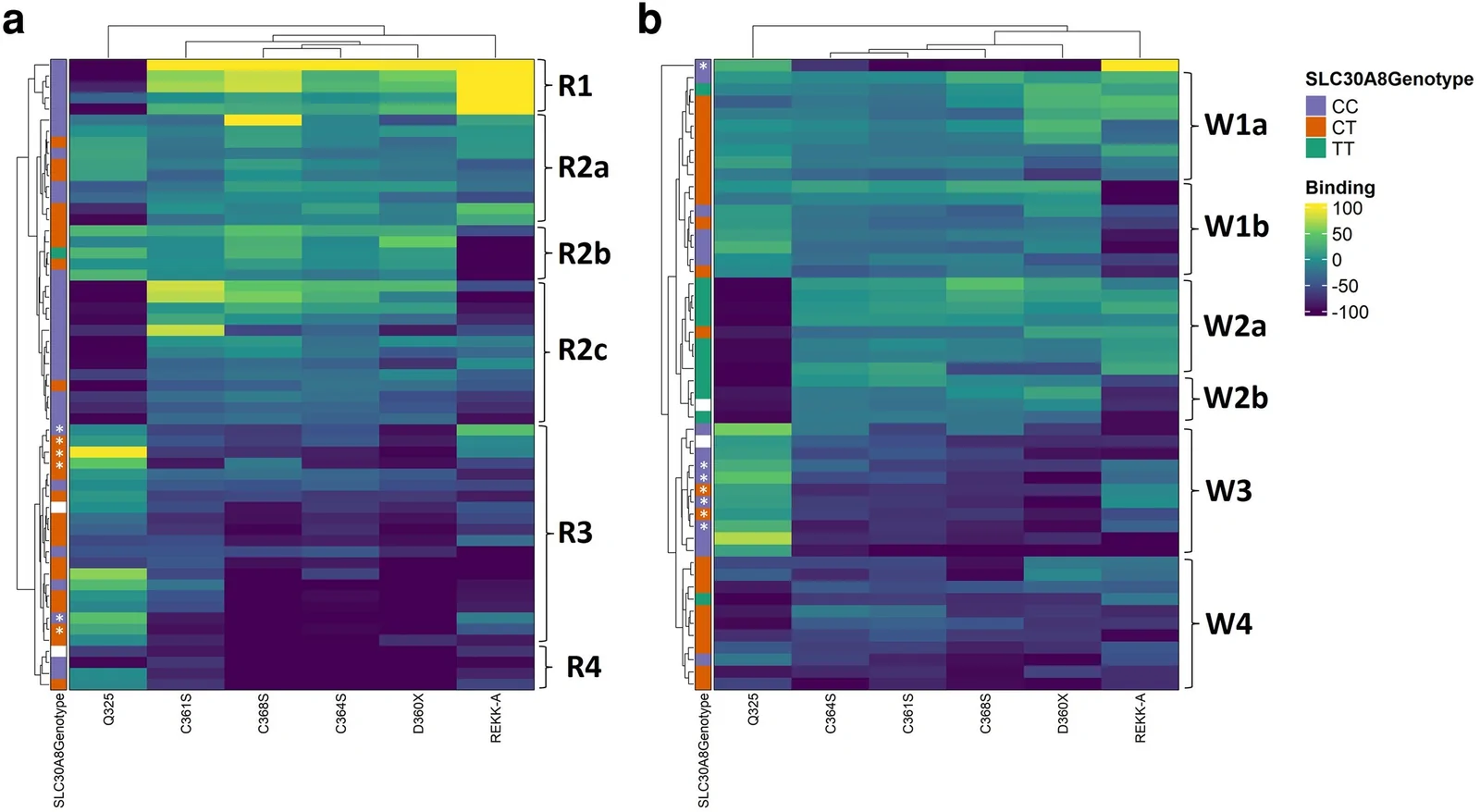
Unbiased Heatmap clustering analysis of reduction in ZnT8A binding segregated by R235-reactive (a) and W325-reactive (b) responses across all ZnT8 mutant antigens with *SLC30A8* genotype denoted. Green to yellow represents subjects with little to no change and increased ZnT8A binding (indexed) compared with wild type ZnT8 antigen. Blue to dark blue represents subjects with reduced ZnT8A binding compared with wild type ZnT8 antigen >−25%. Each individual row denotes a subject’s response across all investigated ZnT8 mutant antigens. Two white squares in *SLC30A8* genotypes denote individuals without a genetic sample available. (a) In R325-positive responses (*n* = 57), three main clusters (x-axis) across ZnT8 mutant antigens, Q325, REKK-A, and the cysteine region (C361S, C364S, C368S, and D360X) and 6 (y-axis) clusters of samples with similar binding patterns (R1, R2a, R2b, R2c, R3, and R4). (b) In W325-positive responses (*n* = 52), similarly three main clustered were observed, Q325, following by REKK-A and the cysteine region, with 6 clusters of samples with similar binding patterns (W1a, W1b, W2a, W2b, W3, and W4). White asterisks (*n* = 6 for both R325-positive and 7 for W325-positive ZnT8A responses) indicate samples where the impact of cysteine region appears largely independent of other major ZnT8A epitopes, R325/W325 or REKK. This analysis showed that all three regions of C-terminal ZnT8 investigated form independent epitopes for ZnT8A binding, with evidence of heterogeneity across subjects. However, in the majority of subjects one or more investigated ZnT8 mutants almost abolished ZnT8A binding to C-terminal ZnT8.

In both heat maps generated, the x-axis represents clustering of ZnT8A binding towards mutant ZnT8 and, the y-axis represents clustering of each sample from the 71 newly diagnosed T1D BOX cohort. In R325-positive ZnT8A responses (*n* = 57), three major clusters were observed, Q325, REKK-A, and the cysteine ZnT8 mutants (C361S/C364S/C368S/C360T) (Fig. 4a), reflecting the three epitope regions studied R325/W235/Q325, REKK, and the C-terminal cysteines, respectively. This was also consistent in W325-positive (*n* = 52) ZnT8A responses (Fig. 4b). On the y-axis, sample clustering most clearly reflected the impact of Q325, with evidence of subjects clustering by *CC* and *TT SLC30A8* genotypes across all ZnT8 mutants.

For both R325-positive and W325-positive ZnT8A responses, clusters of samples with comparable binding were similar, but the order of these clusters varied. (i) Clusters R1 (*n* = 5) and W2a (*n* = 8) were most impacted by Q325, which were enriched with *CC* and *TT SLC30A8* genotypes, respectively. Samples in R2c (*n* = 13) and W2b (*n* = 4) were similar but showed some reduction in both Q325 and REKK-A. (ii) Cluster R2b (*n* = 5) and W1b (*n* = 8) were clusters most impacted by REKK-A. (iii) Cluster R2a (*n* = 10) and W1a (*n* = 9) were generally not negatively impacted by any mutation, although two samples in Cluster R2a were heavily impacted by Q325. (iv) Cluster R4 (*n* = 4) and W4 (*n* = 11) showed reductions across all mutations investigated. (v) Clusters R3 (*n* = 20) and W3 (*n* = 11) were impacted by REKK-A and the cysteine mutations, but Q325 had less impact. These groups included six samples in both R325- positive [6 of 57 (10.5%)] and W325-positive [6 of 52 (11.5%)] ZnT8A responses where binding was the most reduced for the cysteine mutations independent of the other major epitopes (indicated by asterisks in Fig. 4a and b). In addition, one sample for W325 clustered separately to all others, which seemed to be reduced by C-terminal mutations alone [indicated by top asterisk; 7 of 53 (13.2%)].

Overall, at least one cysteine region mutation nearly abolished ZnT8A binding (>75% reduction in binding) in between 12.3 and 36.8% of 57 R325-ZnT8 positive samples (*n* = 7 C361S, *n* = 16 C364S, *n* = 17 C368S, and 21 D360X) and 7.7–28.8% of 53 W325-positive samples (*n* = 4 C361S, *n* = 7 C364S, *n* = 15 C368S, and 12 D360X).

## Discussion

In this study, we conducted a comprehensive investigation of ZnT8A that confirmed findings of the two previously identified major epitopes but also showed that the C-terminal ZnT8 cysteine residues influence ZnT8A binding, and that the human-specific cysteine-rich region is a key element of a relevant additional epitope.

The conformational structure of C-terminal ZnT8 is crucial for ZnT8A recognition and measurement in T1D [1, 10, 13, 14]. Numerous alternative experimental approaches have been used to interrogate autoantibody epitopes such as SDM of recombinant islet antigens (as in the present study), monoclonal antigens [31–33], and 3D modelling. Until recently [20], the 3D structure of the distantly related prokaryotic (*E. coli*) cation diffusion facilitator YiiP protein has been used to model ZnT8. However, YiiP shares only ∼39.5% homology with human ZnT8 [10, 34–36]. Nevertheless, YiiP modelling was used to design the SDM experimental strategy that identified the conformational REKK epitope [10]. After exploring the differences between human and murine ZnT8 (∼80% homology), it was demonstrated that collectively, but not individually, modifying the murine REKK region (T332, G333, Q336, 340 deletion (TGQ-) to human REKK, lead to restored ZnT8A binding in human sera using a comparable *in vitro* C-terminal ZnT8 antigen and RBA method to the present study. This effect was observed regardless of the presence of R325 or Q325. These findings further supported that ZnT8A recognizes conformational epitopes, which was measured in an *in vitro* system, and that the conformational REKK region formed a ZnT8A epitope independent of the polymorphic 325 site, but W325 was not investigated [10].

In the present study, we sought to extend this analysis using a SDM approach in the human C-terminal ZnT8 antigen to consider all human ZnT8A specificities, and showed that 325-agnostic ZnT8A responses to R325-ZnT8, W325-ZnT8, and Q325-ZnT8 antigens were comparably impacted by substituting REKK to AAAA. We also showed that both R325-specific and W325-specific ZnT8A were not substantially impacted by the REKK-A mutation, confirming that the polymorphic epitopes are largely independent of the conformational REKK epitope with the inclusion of W325-specific responses. In addition, we created a human C-terminal antigen construct that encoded the murine TGQ- at this region to better understand the structural impact of the AAAA mutations. The binding of ZnT8A towards mutated REKK (AAAA) was comparable to when the equivalent murine TGQ- was present (data not shown). However, it cannot be ruled out that the TGQ- and AAAA may have different structural consequences allowing/inhibiting ZnT8A accessibility.

For the first time, we provide data on the interaction between ZnT8A and the cysteine-rich region of human C-terminal ZnT8. This area is relevant because the human-specific CXXC motif (C361XXC364) of ZnT8 shows high binding affinity for zinc by cryo-EM [19–21], although the impact of zinc on C-terminal ZnT8 configuration is not known *in vitro* settings. Using SDM, we introduced cysteine-to-serine mutations as well as completely removed the cysteine region using an antigen truncated at aa360 (D360X), and investigated the impact on ZnT8A binding by RBA. This strategy was previously used successfully by our research group for investigating the core/cytosolic cysteines in the intracellular domains of IA-2 (IA-2ic) for IA-2A/IA-2βA binding in newly diagnosed T1D [15]. Our data show that mutation of cysteines in this region reduced ZnT8A binding in 325-agnostic responses (MRB of at least −30% up to −66.4%) and reduced ZnT8A binding below that of the R325W and REKK major epitopes in around 10.5% of subjects with R325-ZnT8 positive ZnT8A and 13.2% of subjects with W325-ZnT8 positive ZnT8A. Moreover, mutating the cysteine region nearly abolished binding (>75% reduced) in 7.7%-36.8% of R325-ZnT8/W325-ZnT8 positive 325-agnostic ZnT8A. Therefore, altering the cysteine region caused a clear decrease of ZnT8A responses in at least 10% of positive subjects, a reduction that appears largely independent of the major epitopes (REKK and R325W).

A strength of the present study, based on well-characterized subjects from a newly diagnosed T1D cohort [22], is that ZnT8A binding to the cysteine region was comprehensively analysed in conjunction with previously identified major C-terminal epitopes in all subjects for the first time [9, 10]. The use of a properly formulated index, using both R325-specific/W325-specific and 325-agnostic controls, in the present study allowed us to evaluate ZnT8A binding to wild-type and mutant C-terminal ZnT8 antigens produced *in vitro*, whilst considering experimental variation and the alterations to the biophysical properties and folding efficiencies that the mutations likely induce. This is a more robust statistical approach than previous similar studies in T1D [1, 10, 15], which mostly relied on using residual radioactivity of immunoprecipitates (cpm). We evaluated ZnT8A specificity for all wild-type ZnT8 variants (R325/W325/Q325) in all analyses, using both *SLC30A8* genotype and ZnT8A binding to polymorphic monomeric R325-ZnT8/W325-ZnT8/Q325-ZnT8 RBAs. We were unable to directly compare the impact of cysteine mutations in R325-specific/W325-specific and 325-agnostic ZnT8A responses because different control sera were available for indexing. Whilst the significance threshold was not met, there was some impact of the C-terminal cysteine mutations in some R325-specific/W325-specific sera which suggests that either these sera are not truly 325-specific or that the cysteine mutants cause a wider conformational change in the *in vitro* C-terminal ZnT8 protein that influences the accessibility or stability of ZnT8A binding to the polymorphic site. However, if the latter was the case, all R325-specific/W325-specific sera would be impacted similarly, but a range of effects were observed. Similarly, in all cysteine mutations, a range of effects between sera were observed in 325-agnostic ZnT8A indicating that the cysteines may differentially impact ZnT8A binding to other epitopes (directly or indirectly). A future avenue of this would be to test sera through competitive displacement, specifically in the cysteine region, to see if ZnT8A binding could be blocked. This would confirm/deny whether the cysteines form a linear epitope, contribute to conformational ZnT8A epitope(s), or simply help to maintain antigen stability and integrity to permit ZnT8A recognition and measurement.

A limitation of this study is that we were unable to examine how modifying the cysteine or REKK regions can influence the tertiary structure of full-length ZnT8 protein (aa1-369) and by extension, ZnT8A binding. This could be done either through molecular modelling of whole human ZnT8 or modifying the proteoliposome-based full-length ZnT8 mature protein model, proposed by Wan *et al.* [37], and would be an interesting future direction of this work. To understand how REKK and cysteine mutations may impact the tertiary structure of ZnT8, we sought to use the modern AI-enhanced structural prediction modelling, AlphaFold 3 [38–40], to model human full-length ZnT8 (R325 or W325; UniProtKB: Q8IWU4.2) in its *in vivo* dimeric configuration. AlphaFold 3 suggested that REKK mutations (REKK-A or murine TGQ-) and cysteine mutations (C361S/C364S/C368S/D360X) do not grossly impact the tertiary structure of dimeric human ZnT8 (data not shown). Although unsurprisingly, AlphaFold 3 predicted that the cysteine mutations are likely to affect the zinc binding site involving C361/C364, and the C-terminal/N-terminal interactions between the two monomers. This could alter the accessibility/binding of ZnT8A towards ZnT8 epitopes *in vivo*. However, this cannot explain our present findings using monomeric ZnT8 peptides that have been expressed *in vitro* (in a reducing environment that prohibits formation of disulphide bonds). The *in vitro* transcription/translation assay system employed in all conventional RBAs is limited by lacking post-translational modifications of recombinant antigens. Conversely, it does not require the addition of tags or reporters that might impact the 3D structure of critical epitopes recognized by humoral responses. However, it is worth noting that the structural configuration of C-terminal ZnT8 (aa268-369) generated *in vitro,* like in the present study, is not known, but has been assessed through detectable antigen-antibody complexes, which can be detected in 60–80% of newly diagnosed T1D sera depending on age [1, 3].

It is not clear why the double cysteine mutations (C361S/C364S and C361S/C368S), did not negatively impact ZnT8A binding to R325-ZnT8 when studied in a subset of individuals. Perhaps intermolecular bonds between the two serine amino acids in these double mutations compensate to stabilize protein structure and/or critical epitope formation for ZnT8A recognition. Cysteine residues are usually substituted for serine due to their isosteric compatibility, though serine is more hydrophobic than cysteine. However, the cysteines are located within the cytosolic domain and not the hydrophobic core of ZnT8; this should be a well-tolerated substitution. The observation that the double cysteine/serine mutations did not impair ZnT8A binding argues against hydrophobicity being the reason the binding is impaired in the single cysteine-to-serine mutants. Previously, we observed that both C909S and C909A within the intracellular region of IA-2ic similarly impaired IA-2A binding [15].

While the focus of this study was to understand ZnT8A epitopes in relation to ZnT8A measurement, we have also observed that a 1% concentration of the non-ionic surfactant, Tween-20, negatively impacted the binding of ZnT8A. These findings extend our prior observations that Tween-20 can affect T1D autoantibody binding in a manner that depends on the antigen-antibody interaction, exhibiting a negative impact on IA-2A, but not on GADA, and on the contrary, has a beneficial effect on IAA measurement [16, 41, 42]. Perhaps this could be related to the fact that both ZnT8 and IA-2 are transmembrane proteins. In particular, for IA-2A, we previously evaluated the importance of specific core cysteines by alkylation of these residues and assessed the modification of the composition of buffers used in conventional RBAs [16] including, in addition to Tween-20, the antibacterial sodium azide and the sulfhydryl modifying agent N-ethyl maleimide. The mechanism by which Tween-20 modifies specific antibody-antigen binding needs further investigation. Whilst it has been postulated Tween-20, as a detergent, can modify cysteines, Tween-20 appears to have more widespread effects on protein stability and integrity, depending on the specific protein and application. It is also possible that Tween-20 could displace weaker antigen-antibody complexes. Low affinity autoantibody responses could be particularly vulnerable to increasing assay detergent concentrations. High-affinity islet autoantibody responses have been shown to be more disease-specific, allowing for improved risk prediction modelling, although detailed characterization of ZnT8A affinity is limited [43]. Whether modifying Tween-20/detergent conditions could discriminate high from low affinity ZnT8A responses warrants further study. Nevertheless, Tween-20 is a common constituent of immunoassay buffers and widely used to reduce protein aggregation and non-specific protein–protein interactions, with the aim to improve sensitivity and specificity. Therefore, differences in reagent constituents might explain at least some of the differences observed in ZnT8A assay performance between laboratories and methods [18, 44, 45]. Since novel methods for measuring ZnT8A [43, 45, 46], alone or multiplexed in assays detecting more than one islet autoantibody [47–49], are being developed and are increasing in popularity, it is important that these experimental strategies adopt antigen constructs preserving the ZnT8 C-terminal cysteine region and carefully evaluate the buffer composition.

Together with the essentially confirmatory results we obtained for the non-polymorphic conformational epitope REKK and the polymorphic R325W site, our analysis revealed the remarkable heterogeneity and complexity of mature ZnT8A responses towards the cytosolic C-terminal region of ZnT8 in newly diagnosed T1D, and why ZnT8A specificity should be considered in future studies and assay development strategies. Through unbiased hierarchical cluster analysis, we were able to demonstrate the heterogeneity of mature ZnT8A responses directed towards the C-terminal of ZnT8 in newly diagnosed T1D and that the ZnT8A epitope regions are independent of each other. This likely indicates that ZnT8 is differentially presented to the immune system between individuals, which does not appear related to *SLC30A8* genotype or level. Whilst the primary epitope of mature ZnT8A close to T1D diagnosis is the C-terminal of ZnT8 [1], characterization of the natural history of ZnT8A responses during the prodrome of T1D is limited. There is increasing evidence that ZnT8A may appear earlier than primary responses (IAA/GADA [50]), in a small cohort of genetically high-risk children, when using a ZnT8 antigen construct that combines the C-terminal with transmembrane segments of ZnT8 that are extracellularly exposed [51, 52]. This may indicate that other/additional ZnT8 epitopes are presented to the immune system early in T1D autoimmunity, which would have highly important connotations for early detection of pre-clinical T1D autoimmunity.

## Conclusion

In summary, we have shown that the three cysteines contained in the last nine amino acids of ZnT8 contribute to a minor epitope for ZnT8A binding. These cysteines may be impacted by detergents commonly added to immunoassays and have effects independent of the previously identified epitopes of ZnT8A. ZnT8A remains an essential part of efficient strategies to identify islet autoantibodies in general population cohorts. Allowing the prevention of DKA and identification of high-risk individuals for clinical trials and novel therapies [53]. Several ZnT8A test formats have been successful including ELISA, RBA, LIPS, and electrochemiluminescence. However, these assays have never been fully harmonized and recent IASP data [54] and data from the ENDIA study [55], suggest that there are clear differences (e.g. low-risk ELISA positives not confirmed by RBA or LIPS) that require formal investigation. In addition, the clinical utility of ZnT8A requires innovative solutions to reduce cost and improve efficiency of autoantibody testing, not least through point of care tests. This study outlines the complexity of ZnT8A as a biomarker and should provide assay developers with valuable data. The epitope spreading of ZnT8A during the natural history of T1D remains unclear and requires further investigation in larger studies that are adequately powered to investigate the influence of age and ethnicity on ZnT8A humoral profiles and future T1D risk. These investigations are particularly important in light of emerging efforts to understand pre-T1D within the general population, adults, and diverse ethnicities.

## Supplementary Material

uxag050_Supplementary_Data

## Data Availability

The datasets are available from the corresponding author on reasonable request.

## Abbreviations:

325-agnostic ZnT8A: non-polymorphic zinc transporter 8 autoantibodies
AU: arbitrary units
BOX: Bart’s Oxford (BOX) family study
ELISA: enzyme-linked immunosorbent assay
GADA: glutamic acid decarboxylase 65 autoantibodies
GWAS: genome-wide association study
IA-2A: islet antigen 2 autoantibodies
IAA: insulin autoantibodies
IASP: Islet Autoantibody Standardization Programme
MRB: median reduction in binding
R325-specific ZnT8A: zinc transporter R325 (arginine)-specific autoantibodies
R325W: arginine to tryptophan single nucleotide polymorphism at amino acid 325 of ZnT8 (rs13266634)
RBA: radiobinding assay
REKK: R332 (arginine), E333 (glutamic acid), K336 (lysine), and K340 (lysine) amino acids in C-terminal zinc transporter 8
SDM: site-directed mutagenesis
SNP: single nucleotide polymorphism
T1D: type 1 diabetes
W325-specific ZnT8A: zinc transporter W325 (tryptophan)-specific autoantibodies
ZnT8: zinc transporter 8
ZnT8A: zinc transporter 8 autoantibodies
