# Supplementary material for: The C-terminal cysteines in zinc transporter 8 (ZnT8) form an independent epitope for autoantibody binding in newly diagnosed type 1 diabetes
Source: Clin Exp Immunol. 2026 Aug 31;220(1):uxag050. doi: 10.1093/cei/uxag050 (PMC13537458; doi:10.1093/cei/uxag050)
Supplement: Supplementary file 1 [file uxag050_supplementary_data.pdf]

Wild-type C-terminal ZnT8 (aa368-369)

FASTA

[*atggag*]aaggacttctccatcttactcatggaaggtgtgccaaagagcctgaattacagtggtgtgaaagagcttattttagcagtcgacggggtgctgtctgtgcacagc

ctgcacatctggtctctaacaatgaatcaagtaattctctcagctcatgttgctacagcagccagc**CGG**gacagccaagtggttcgg**AGAGAA**attgct**AAA**gccctta

gc**AAA**agctttacgatgcactcactcaccattcagatggaatctccagttgaccaggaccccgac**TGC**cttttc**TGT**gaagacccc**TGT**gac[*tag*]

Protein Sequence

*me*kdfsillmegvpkslnysgvkelilavdgvlsvhslhiwsltmnqvilsahvataas**R**dsqvvr**RE**ia**K**als**K**sftmhsltiqmespvdqdpD**C**LF**C**EDP**C**D]

Supplementary Figure 1: Template (wild-type) C-terminal ZnT8 DNA sequence (aa268-369) and mutation sites.

Green: Start and stop codons with added glutamate (gag) codon. Red: *SLC30A8* polymorphic SNP site (rs13266634; R325W) encoding the major allele (R325; CGG); minor alleles W325 (TGG; MAF: 0.3) and Q325 (CAG; rs168899462; MAF <0.01 in Europeans) forming one of the major ZnT8A epitopes [1]. Blue: Previously identified conformational ZnT8A epitope R332, E333, K336, and K340 (REKK) [2]. Orange: Three cysteines C361, C364, and C368; C361 and C364 form part of the highly conserved CXXC motif which has been shown to have high affinity for zinc and may aid in the allosteric mechanism of ZnT8 that is required for zinc trafficking and ZnT8's function [3-5]. Scissors and dashed line indicate the location of the truncation at aa360 (D360T).

Mutation	C-terminal amino acid position(s)	Primer Sequences (5' ⟶ 3')
**C361S**	361	F: GACCAGGACCCCGAC**AGC**CTTTTCTGTGAAG (31bp)<br>R: CTTCACAGAAAAG**GCT**GTCGGGGTCCTGGTC (31bp)
**C364S**	364	F: CCCGACTGCCTTTTC**AGT**GAAGACCCCTGTG (31bp)<br>R: CACAGGGGTCTTC**ACT**GAAAAGGCAGTCGGG (31bp)
**C368S***	368	F: TTTTCTGTGAAGACCCC**AGT**GACTAGGAATTCACG (35bp)<br>R: CGTGAATTCCTAGTC**ACT**GGGGTCTTCACAGAAAA (35bp)
**C361S/C364S**	361 & 364	F: CCCGAC**AGC**CTTTTC**AGT**GAAGACCCCTGTG (31bp)<br>R: CACAGGGGTCTTC**ACT**GAAAAG**GCT**GTCGGG (31bp)
**C361S/C368S***	361 & 368	N/A
**R325-Q325**	325	F: ACAGCAGCCAGC**CAG**GACAGCCAAGTG (27bp)<br>R: CACTTGGCTGTC**CTG**GCTGGCTGCTGT (27bp)
**R325-W325**	325	F: CTACAGCAGCCAGC**TGG**GACAGCCAAGTG (29bp)<br>R: CACTTGGCTGTC**CCA**GCTGGCTGCTGTAG (29bp)
**E333A**	333	F: CCAAGTGGTTCGGAGA**GCA**ATTGCTAAAGCCCTTA (35bp)<br>R: TAAGGGCTTTAGCAAT**TGC**TCTCCGAACCACTTGG (35bp)
**E333A/K336A**	333 & 340	F: **CA**ATTGCTAAAGCCCTTAGC**GCA**AGCTTTACGATGCACTCAC (42bp)<br>R: GTGAGTGCATCGTAAAGCT**TGC**GCTAAGGGCTTTAGCAAT**TG** (42bp)
**R332A/E333A/K336A**	332, 333 & 340	F: ACAGCCAAGTGGTTCGG**GCAGCA**ATTGCTAAAGCCC (36bp)<br>R: GGGCTTTAGCAAT**TGCTGC**CCGAACCACTTGGCTGT (36bp)
**R332A/E333A/K336A/K340A (REKKA)**	332, 333, 336 & 340	F: GTGGTTCGG**GCAGCA**ATTGCT**GCA**GCCCTTAGC**GC** (35bp)<br>R: **GC**GCTAAGGGC**TGC**AGCAAT**TGCTGC**CCGAACCAC (35bp)
**Murine REKK (T332/G333/Q336/340 deletion; TGQ-)**	332, 333, 336 & 340	F: GCACTCGAGAATTCGCCGCCACCATGGAGA (30bp)<br>R: TAAAGCTT**TGA**AAGGGC**CTG**AGCAAT**TCCCGT**CCGAACCACTTGGCTGT (52bp)

Supplementary Table 1: Primer sequences of C-terminal ZnT8 mutations

N/A: Not applicable; F: Forward primer; R: Reverse primer. Serine or alanine amino acid substitutions was conducted by SDM using primers designed by Agilent (Santa Clara, USA) QuikChange II SDM Instruction Manual and were PAGE-purified at 0.05pmol/µl (Sigma, St. Louis, Missouri, United States). Bold denotes the location of the amino acid mutation(s). The generation of a ZnT8 construct encoding murine REKK (TGQ-) was conducted through molecular cloning strategies: red denotes the location of amino acid deletion at aa340; dark grey highlight denotes the *XhoI* restriction enzyme recognition site sequence; light grey denotes the *HindIII* restriction enzyme recognition site sequence.* To generate the ZnT8 construct with the double C361S/C368S mutation, SDM was conducted using the ZnT8 construct with C361S as template DNA and C368S forward and reverse primers. The truncation at aa360 (D360T) was generated by V. Lampasona's laboratory (Milan, Italy); information on the primers and experimental design conducted was unavailable.

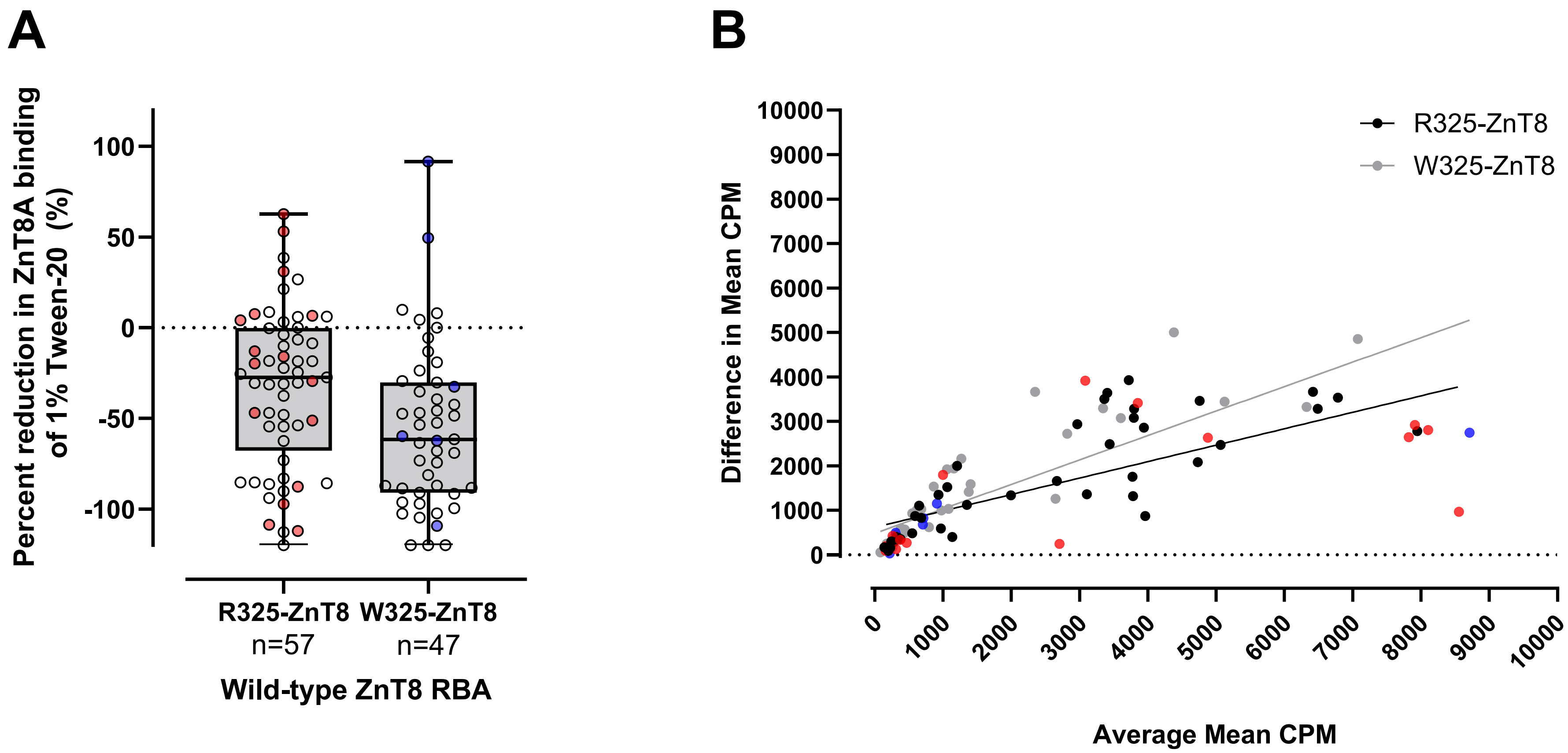

Supplementary Figure 2: The effect of high (1%) and low (0.1%) Tween-20 Concentrations in TBST assay buffer on ZnT8A binding towards wild-type ZnT8-R325 and ZnT8-W325 in monomeric RBAs.

(A) Percent reduction in ZnT8A binding (indexed to 325-agnostic sera) of using a 1% Tween-20 concentration TBST assay buffer compared to 0.15% (standard) in SWENDIC ZnT8A positive sera (n=63) by R325-ZnT8 and W325-ZnT8 RBAs. Black dotted line: no change in ZnT8A binding compared with wild-type ZnT8 antigen (R325-ZnT8 or W325-ZnT8) by RBA. Filled in circles indicates R325-specific (red; n=16; -17.9%) and W325-specific (blue; n=6; -46.2%) ZnT8A responses and open circles indicate 325-agnostic ZnT8A (n=41; -30.3% using R325-ZnT8 and -63.6% using W325-ZnT8). Boxes and whisker plots represent median, interquartile ranges, and range of percent reduction in ZnT8A binding. All ZnT8A binding was reduced using a 1% Tween-20 TBST assay buffer in R325-ZnT8 and W325-ZnT8 RBAs (both $p<0.0001$). The median reduction in ZnT8A binding was -27.4% in R325-positive and -61.6% in W325-positive ZnT8A. In 325-agnostic ZnT8A responses, ZnT8A binding to wild-type W325-ZnT8 antigen was lower than binding to R325-ZnT8 antigen ($p<0.0001$). There was a wide range of effect observed from minimal reduction to almost abolished ZnT8A binding [range +91.6 to -136% (truncated on graphs for improved resolution)]. **(B)** Scatterplot of the difference in mean CPM versus the average CPM. Black and Grey lines denote the linear regression lines for R325-ZnT8 and W325-ZnT8 positive ZnT8A by RBA; R325-ZnT8 $Y = 0.3698*X + 614.7$ (R2: 0.5248) and W325-ZnT8 $Y = 0.5500*X + 481.7$ (R2: 0.7007), both $p<0.0001$. Red filled in circles indicate R325-specific ZnT8A, blue filled in circles indicate W325-specific ZnT8A, and black/grey filled in circles denote aa325-agnostic ZnT8A in R325-ZnT8/W325-ZnT8 RBAs, respectively. This plot highlights that the reduction in ZnT8A binding can be observed across all levels of ZnT8A from low to high level positivity.

Wild-type ZnT8 antigen	ZnT8A specificity	C361S *r* (95% CI) *p value*	C364S *r* (95% CI) *p value*	C368S *r* (95% CI) *p value*	D360X *r* 95% CI) *p value*
**R325**	**R325-specific** n=12	0.88 (0.61-0.97) 0.0003	0.95 (0.82-0.99) <0.0001	0.90 (0.67-0.97) 0.0002	0.92 (0.73-0.98) <0.0001
**R325**	**325-agnostic** n=45	0.90 (0.83-0.95) <0.0001	0.93 (0.87-0.96) <0.0001	0.88 (0.79-0.94) <0.0001	0.91 (0.83-0.95) <0.0001
**W325**	**W325-specific** n=13	0.98 (0.94-1.00) <0.0001	0.97 (0.89-0.99) <0.0001	0.91 (0.72-0.97) <0.0001	0.90 (0.67-0.97) <0.0001
**W325**	**325-agnostic** n=39	0.96 (0.93-0.98) <0.0001	0.94 (0.89-0.97) <0.0001	0.85 (0.72-0.92) <0.0001	0.90 (0.81-0.95) <0.0001

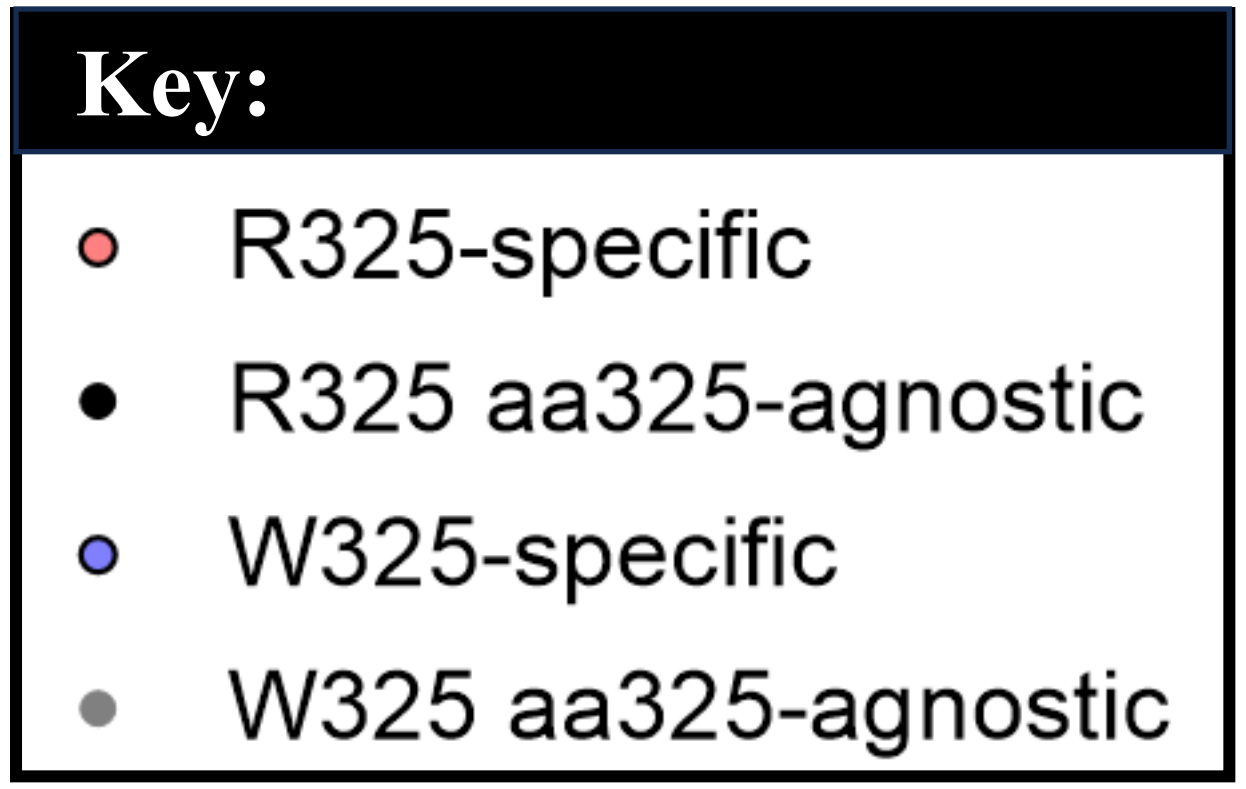

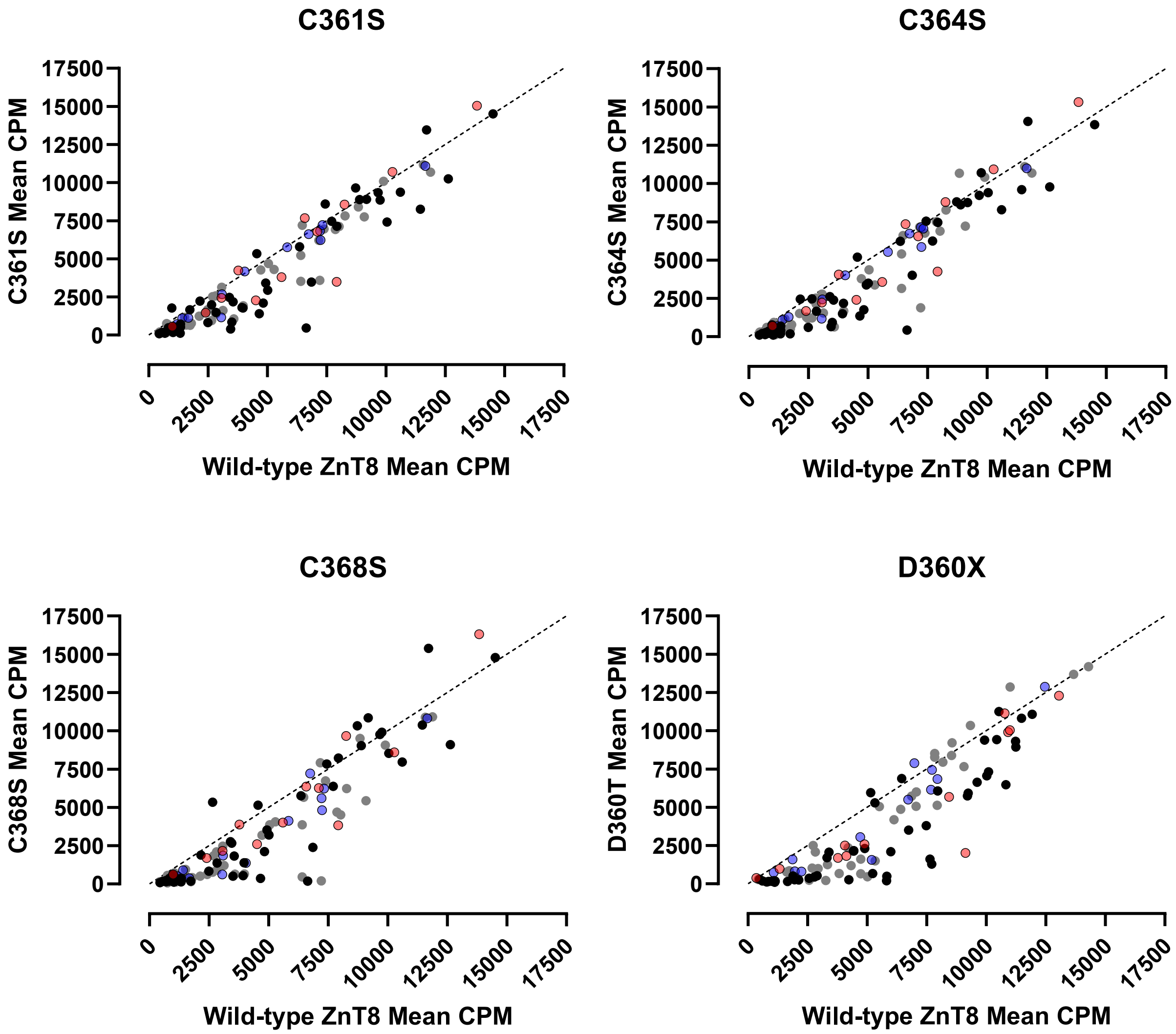

Supplementary Figure 3: ZnT8A binding (mean CPM) between single cysteine-to-serine mutant and wild-type ZnT8 antigens was highly correlated, irrespective of ZnT8A specificity.

Binding of ZnT8A, irrespective of ZnT8A specificity, to single cysteine-to-serine mutations (C361S, C364S, and C368S) and truncation at D360 (D360T) was highly correlated with ZnT8A binding to wild-type R325-ZnT8 and W325-ZnT8 antigens (all p<0.0001; Spearman's *r* range: 0.85-0.98).

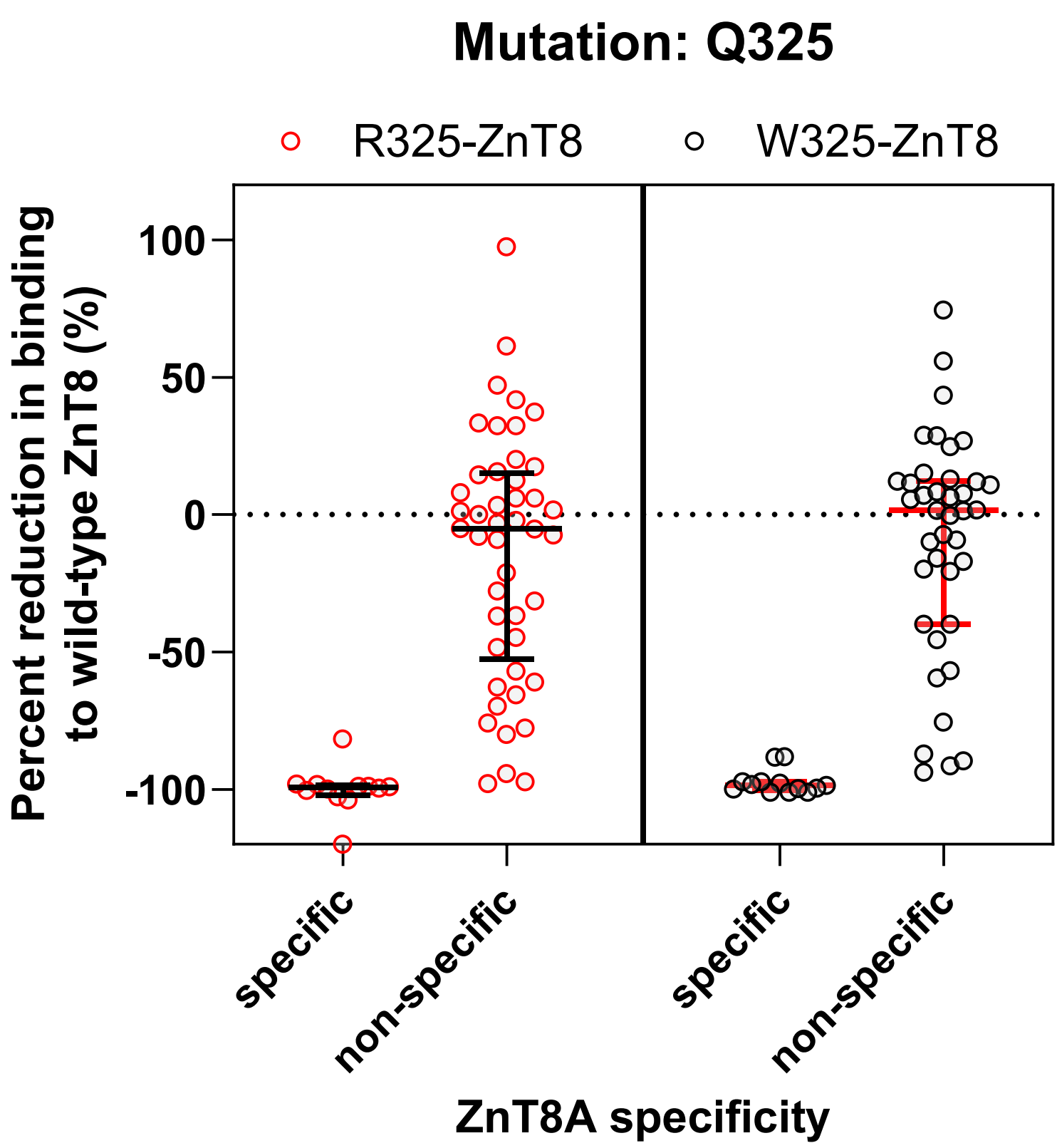

ZnT8 wild-type antigen	ZnT8A Specificity	n	Median reduction (%) in binding to wild-type ZnT8 (range)	*P* value*
R325-ZnT8	R325-specific	12	-99.3 (-144.8 to -81.3)	0.0005
R325-ZnT8	325-agnostic	45	-5.1 (-97.9 to +97.6)	NS
W325-ZnT8	W325 specific	13	-98.5 (-101.1 to -88.1	0.0002
W325-ZnT8	325-agnostic	39	+1.7 (-93.8 to +74.5)	NS

Supplementary Figure 4: The effect of the Q325 mutation on ZnT8RA & ZnT8WA binding according to overall ZnT8A specificity in new-onset type 1 diabetes

NS: Not significant. Black dotted line: no change in ZnT8A compared with wild type (WT) ZnT8 antigen. Red/black bars denote median and interquartile ranges. * Wilcoxon signed-rank test was used to evaluate change from zero in ZnT8A binding. One-way Kruskal-Wallis test with the Dunn post-hoc test was used to compare the median percent reduction in ZnT8A binding (MRB) between categories. The Q325 mutation caused significant reductions in binding in R325-specific ($p=0.0005$) and W325-specific ($p=0.0002$) ZnT8A responses, but not 325-agnostic responses ($p>0.05$). The MRB demonstrates that Q325 abolishes (≥-90%) ZnT8A binding in R325-specific (MRB -99.3%) and W325-specific (MRB -98.5%) responses, compared to 325-agnostic responses to either R325-ZnT8 (MRB -5.1%) or W325-ZnT8 (MRB +1.7%) antigens, clearly demonstrating that these individuals are heavily reliant on the major epitopes at the polymorphic SNP site (aa325).

		SLC30A8 Genotype (rs13266634)			p value
		CC	CT	TT	
	n (69)	30 (43.5)	27 (39.1)	12 (17.4)	
All variants	35 (50.7)*	14 (40.0)	20 (57.1)	1 (2.9)	0.0006
R325-reactive	55 (79.7)*	30 (54.5)	24 (43.6)	1 (1.8)	6.73×10^{-11}
W325-reactive	50 (72.5)*	15 (30.0)	23 (46.0)	12 (24.0)	0.0008
Q325-reactive	43 (62.3)*	18 (41.9)	23 (53.5)	2 (4.7)	0.0002
R325-reactive only	12 (17.4)	11 (91.7)	1 (8.3)	0 (0.0)	0.013§
W325-reactive only	13 (18.8)	0 (0.0)	3 (23.1)	10 (76.9)	0.0002§
R325/W325-reactive	1 (1.4)	1 (100.0)	0 (0.0)	0 (0.0)	¥
R325/Q325-reactive	7 (10.0)	4 (57.1)	3 (42.9)	0 (0.0)	¥
W325/Q325-reactive	1 (1.4)	0 (0.0)	0 (0.0)	1 (100.0)	¥

Supplementary Table 2: ZnT8A specificity to wild type ZnT8 according to *SLC30A8* genotype

Data are presented as n (%) unless otherwise stated. Of 71 new-onset T1D patients, 69 had *SLC30A8* genotype data. * Categories with 1 missing data set. Data were analysed by χ2 or Fisher's exact tests [§; comparing genotypes containing the C allele (CC/CT) and T allele (TT/CT)] where indicated]. ¥ Categories with frequencies less than 5 could not be compared.

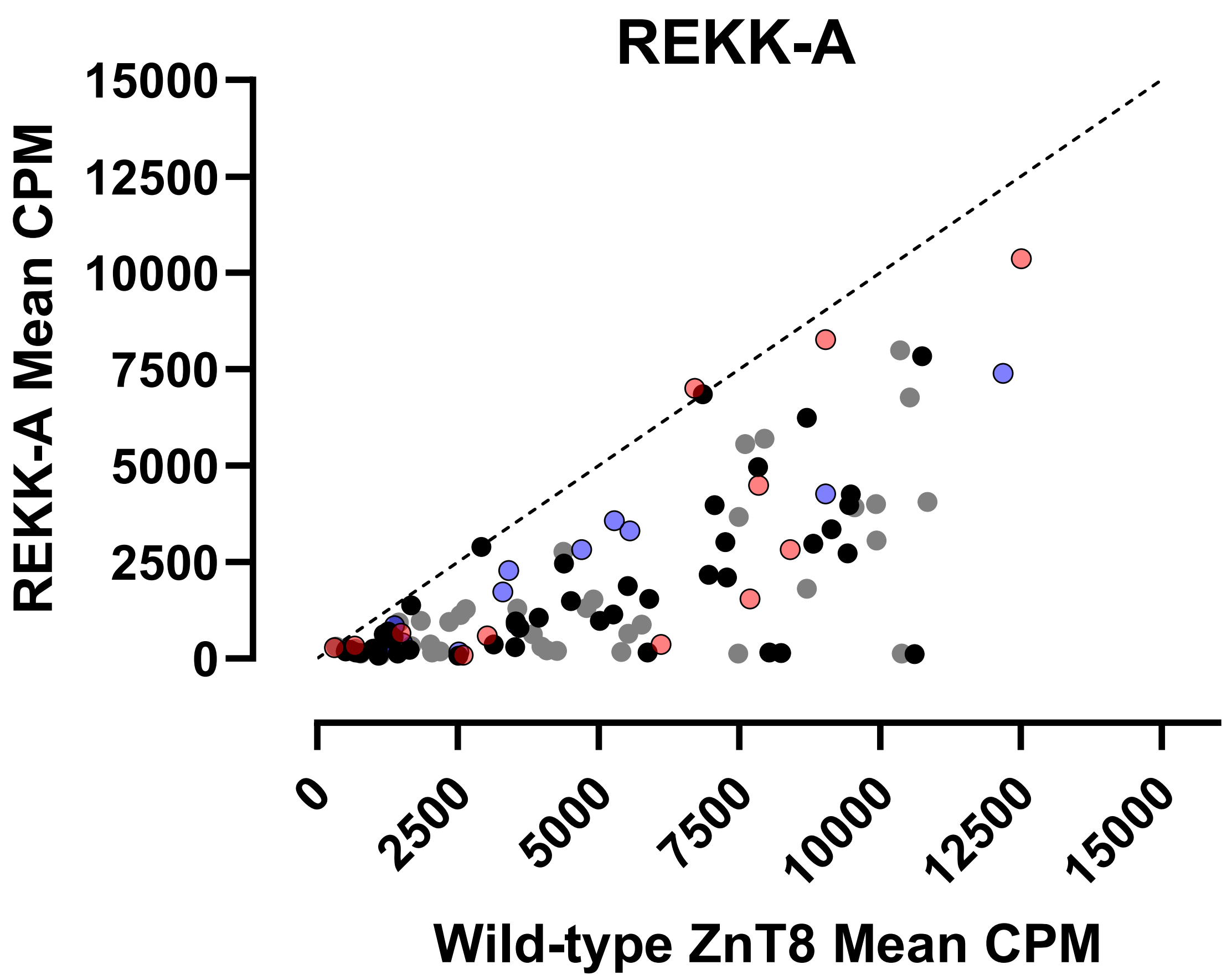

Key:

- R325-specific
- R325 aa325-agnostic
- W325-specific
- W325 aa325-agnostic

Wild-type ZnT8 antigen	ZnT8A specificity	REKK-A *r* (95% CI)
R325	R325-specific n=12	0.87 (0.57-0.96) 0.0005
R325	325-agnostic n=45	0.60 (0.37-0.76) <0.0001
W325	W325-specific n=13	0.92 (0.73-0.98) <0.0001
W325	325-agnostic n=39	0.59 (0.33-0.77) <0.0001

Supplementary Figure 5: ZnT8A binding (mean CPM) between REKK-A mutant and wild-type ZnT8 antigens was highly correlated, irrespective of ZnT8A specificity.

Binding of ZnT8A, irrespective of ZnT8A specificity, to REKK-A was highly correlated with ZnT8A binding to wild-type R325-ZnT8 and W325-ZnT8 antigens ($p=0.005$ to $p<0.0001$; Spearman's r range: 0.59-0.92).

References for Supplementary Material:

1. Williams CL, Long AE. What has zinc transporter 8 autoimmunity taught us about type 1 diabetes? *Diabetologia.* 2019;62(11):1969-76.

2. Wenzlau JM, Frisch LM, Hutton JC, Davidson HW. Mapping of conformational autoantibody epitopes in ZNT8. *Diabetes Metab Res Rev*. 2011;27(8):883-6.

3. Parsons DS, Hogstrand C, Maret W. The C-terminal cytosolic domain of the human zinc transporter ZnT8 and its diabetes risk variant. *FEBS J*. 2018;285(7):1237-50.

4. Xue J, Xie T, Zeng W, Jiang Y, Bai XC. Cryo-EM structures of human ZnT8 in both outward- and inward-facing conformations. *Elife*. 2020;9.

5. Catapano MC, Parsons DS, Kotuniak R, Mladenka P, Bal W, Maret W. Probing the Structure and Function of the Cytosolic Domain of the Human Zinc Transporter ZnT8 with Nickel(II) Ions. *Int J Mol Sci*. 2021;22(6).

6. Wenzlau, J.M., Y. Liu, L. Yu, O. Moua, K.T. Fowler, S. Rangasamy, J. Walters, G.S. Eisenbarth, H.W. Davidson, and J.C. Hutton. A common nonsynonymous single nucleotide polymorphism in the SLC30A8 gene determines ZnT8 autoantibody specificity in type 1 diabetes. *Diabetes*, 2008. **57**(10): p. 2693-7.
